# The Elite Alleles of *OsSPL4* Regulate Grain Size and Increase Grain Yield in Rice

**DOI:** 10.1186/s12284-021-00531-7

**Published:** 2021-11-02

**Authors:** Jihong Hu, Liyu Huang, Guanglong Chen, Hui Liu, Yesheng Zhang, Ru Zhang, Shilai Zhang, Jintao Liu, Qingyi Hu, Fengyi Hu, Wen Wang, Yi Ding

**Affiliations:** 1 State Key Laboratory of Hybrid Rice, College of Life Sciences, Wuhan University, Wuhan, 430072 China; 2 State Key Laboratory of Genetic Resources and Evolution, Kunming Institute of Zoology, Chinese Academy of Sciences, Kunming, 650223 China; 3 School of Ecology and Environment, Northwestern Polytechnical University, Xi’an, 710072 China; 4 State Key Laboratory for Conservation and Utilization of Bio-Resources in Yunnan, School of Agriculture, Yunnan University, Kunming, 650500 China; 5 Germplasm Bank of Wild Species, Kunming Institute of Botany, Chinese Academy of Sciences, Kunming, 650201 China; 6 BGI-Baoshan, Baoshan, 678004 Yunnan China

**Keywords:** *OsSPL4*, Grain size, Grain weight, Panicle branching, Grain yield, Rice

## Abstract

Grain weight and grain number, the two important yield traits, are mainly determined by grain size and panicle architecture in rice. Herein, we report the identification and functional analysis of *OsSPL4* in panicle and grain development of rice. Using CRISPR/Cas9 system, two elite alleles of *OsSPL4* were obtained, which exhibited an increasing number of grains per panicle and grain size, resulting in increase of rice yield. Cytological analysis showed that *OsSPL4* could regulate spikelet development by promoting cell division. The results of RNA-seq and qRT-PCR validations also demonstrated that several MADS-box and cell-cycle genes were up-regulated in the mutation lines. Co-expression network revealed that many yield-related genes were involved in the regulation network of *OsSPL4*. In addition, *OsSPL4* could be cleaved by the osa-miR156 in vivo, and the *OsmiR156*-*OsSPL4* module might regulate the grain size in rice. Further analysis indicated that the large-grain allele of *OsSPL4* in *indica* rice might introgress from *aus* varieties under artificial selection. Taken together, our findings suggested that *OsSPL4* could be as a key regulator of grain size by acting on cell division control and provided a strategy for panicle architecture and grain size modification for yield improvement in rice.

**Supplementary Information:**

The online version contains supplementary material available at 10.1186/s12284-021-00531-7.

## Introduction

Rice (*Oryza sativa* L.) is a staple food for more than half of the world’s population. Increasing grain yield is a long-term goal for crop breeding to meet the demand of global food security. At the individual plant level, rice grain yield is determined by three component traits: number of tillers (panicles) per plant, number of grains per panicle, and grain weight (Mao et al. 2010). Panicle length, the number of panicle branches and the number of spikelets per panicle are the components of panicle architecture (Bai et al. 2017). In rice, grain weight is controlled by grain size which includes grain length, grain width and grain thickness (Zuo and Li 2014; Hu et al. 2015b). Grain size is a key determinant of grain yield and is also a target trait during domestication and breeding in rice (Xia et al. 2018).

In the past years, many genes and quantitative trait loci (QTLs) controlling panicle architecture and grain size have been cloned and characterized (Bai et al. 2012; Zou and Li, 2014; Li et al. 2019). A few genes for panicle architecture have been well studied, such as *OsGRF6*, *OsSPL14* and *OsLG1*, which could promote panicle branching or form compact panicle (Gao et al. 2015; Miura et al. 2010; Zhu et al. 2013). For grain size, several genes, including *DEP1*, *GS3*, *GL3.1*, *GL7*, *GL3.3*/*TGW3* and *OsGRF4*, have been reported to regulate grain length through coordinating alternation of cell division and expansion (Mao et al. 2010; Qi et al. 2012; Wang et al. 2015; Sun et al. 2016; Xia et al. 2018; Ying et al. 2016). And other genes such as *GW2*, *qSW5/GW5*, *GS5*, *GS6*, *GW7* and *GW8* regulate grain width via activation of cell division (Song et al. 2007; Weng et al. 2008; Li et al. 2011; Wang et al. 2012; Sun et al. 2013; Liu et al. 2017). Plant hormones (brassinosteroid and auxin) have also been reported to directly or indirectly participate in controlling rice grain size (Hu et al. 2018b; Li and Li, 2016). For example, *qGL3/OsPPKL1*, *GS2/GL2*, *GS5* and *GW5* are involved in brassinosteroid (BR) signaling pathway (Weng et al. 2008; Li et al. 2011; Zhang et al. 2012; Che et al. 2015; Liu et al. 2017), while *TGW6* and *BG1* are induced by auxin (Ishimaru et al. 2013; Liu et al. 2015). However, only several genes affect both panicle architecture and grain size, including *OsmiR397*-*OsLAC*, *FZP* and *OsSPL13* (Zhang et al. 2013; Si et al. 2016; Bai et al. 2017). Although a number of QTLs/genes for panicle architecture and grain size have been cloned, more genes are needed to understand the genetic and molecular basis of rice yield. Therefore, more genes controlling these important traits remain to be identified for further improvement of yield in rice.

As a class of plant-specific transcription factors, the SQUAMOSA PROMOTER BINDING PROTEIN (SBP)-like (SPL) family proteins all contain a highly conserved DNA-binding domain (SBP domain) of 76 amino acids, harboring a nuclear localization signal at its C-terminus, which is sufficient to bind DNA contained two zinc-fingers of a unusual structure (Birkenbihl et al. 2005; Xie et al. 2006; Yamasaki et al. 2006). It has been proved that the DNA binding domain (DBD) of SBP-box genes is necessary and sufficient to bind a palindromic GTAC core motif (Birkenbihl et al. 2005; Guo et al. 2008). In addition, some studies have demonstrated that the miR156-SPL module plays important roles in plant growth and development (Wang and Zhang 2017; Dai et al. 2018). Recent studies showed that SPL family genes regulate the panicle architecture or grain size, thus affecting the grain yield in rice (Jiao et al. 2010; Wang et al. 2012; Si et al. 2016). Fine-tuning the expression of *SPL* may provide a strategy for increasing grain productivity in rice breeding (Wang and Zhang 2017).

In rice, a total of 19 *SPL* genes were identified as six subgroups. Many of these genes are located within segmental duplication regions, including the gene pairs of *OsSPL3*/*12*, *OsSPL4/11*, *OsSPL14/17* and so on (Xie et al. 2006; Zhong et al. 2019). Several *OsSPL* genes (*OsSPL13*, *OsSPL14*, *OsSPL16* and *OsSPL18*) have been cloned and reported to regulate panicle development or grain size as well as grain shape and quality in rice (Jiao et al. 2010; Miura et al. 2010; Wang et al. 2012, 2018; Si et al. 2016). On the other hand, *OsSPL8/OsLG1* controls the compact panicle architecture in domesticated rice cultivars (Zhu et al. 2013). Moreover, *OsSPL6* can repress the endoplasmic reticulum (ER) stress signaling outputs to prevent the occurrence of panicle apical abortion (Wang et al. 2018). *OsSPL7* has been reported to regulate tiller number and plant height via miR156f-OsSPL7-OsGH3.8 pathway (Dai et al. 2018). In addition, *OsSPL9* can mediate the transcriptional activation of miR528 and orchestrates the antiviral response in rice (Yao et al. 2019). Lately, *OsSPL3*/*OsSPL12* were reported to directly activate *OsMADS50* in the node, which regulates the crown root development in rice (Shao et al. 2019). In addition, comprehensive functional assessments of the *OsSPL* gene family in rice preliminary revealed some of them regulate grain size with mutants using CRISPR/Cas9 (Jiang et al., 2020). However, the biological function of other *OsSPLs*, including *OsSPL4* in rice is still limited.

*OsSPL4* and *OsSPL11* are a duplicated gene pair, but the last exon of *OsSPL4* is shorter than that of *OsSPL11.* Most of the rice *OsSPL* genes possessed a unique motif which could be targeted by OsmiR156, but the miRNA target site of *OsSPL4* was located in the 3′-UTR, which is similar to *OsSPL13* (Xie et al. 2006; Si et al. 2016). In *Arabidopsis*, the *OsSPL4* homologue genes, *AtSPL2*, *AtSPL10* and *AtSPL11* have been reported to control the development of lateral organs associated with shoot maturation in the reproductive phase (Shikata et al. 2009). Lately, systematic biology analysis revealed that *OsSPL4* might affect panicle development and grain weight to enhance yield in rice (Hu et al., 2020; Jiang et al., 2020). However, the biological function, artificial selection and evolution of *OsSPL4* in rice have not been well characterized.

Here, we reported the molecular and genetic analysis of *OsSPL4* and revealed some important structural and functional features as well as the evolution of the *OsSPL4* in panicle architecture and grain size regulation of rice. Our results suggested that *OsSPL4* plays multiple roles in panicle architecture, grain size and yield in rice.

## Materials and Methods

### Plant Materials and Growth Conditions

The Nipponbare rice (*Oryza sativa* L. subsp. *japonica*. cv. Nipponbare) and different independent homozygous CRISPR/Cas9 mutation lines of T3 generation were used in this study. Meanwhile, the empty vector with Nipponbare background was used as the control. Using CRISPR-P (http://crispr.hzau.edu.cn/CRISPR2/), a 20 bp target site (5′-AGGTGCCAGGTGGAAGGGTG-3′) upstream of the protospacer-adjacent motif (PAM) was chosen for *OsSPL4* (LOC_Os02g07780) gene editing with the CRISPR/Cas9 system. The target vectors were constructed using CRISPR/Cas9 binary vector pCAMBIA1300-OsU3-Cas9 by the restriction enzyme *Aar* I as described (Huang et al. 2018). Among the transgenic plants of the T0 generation, the various mutations of *OsSPL4* were confirmed by sequencing.

To generate transgenic rice plants by overexpressing of *OsSPL4* and OsmiR156, the coding region from first-strand cDNA of *OsSPL4*, and the genomic DNA sequences of pre-miR156 was amplified and cloned, respectively. The PCR product was then inserted into a pBWA vector along with the maize UBIQUITIN (Ubi) promoter and Nos terminator. To efficient degradation of OsmiR156, the short tandem target mimic (STTM) of STTM156k was constructed as previous described (Yan et al. 2012). The primer sequences for these constructs are listed in Additional file 1: Table S1.

The vectors were transferred into EH105, a strain of *Agrobacterium tumefaciens* by electroporation. The calli derived from cultivar Nipponbare rice were used for *Agrobacterium*-mediated transformation (Hiei et al., 1994). The control plants and independent transgenic lines were grown in a paddy field at Jinghong (southern of Yunnan province, 22°01'N/100°49'E) under natural conditions. Field management was performed with standard procedures to prevent yield loss during the growth period. Phenotypic data were collected at the maturing stage.

### Off-Target Detection

The potential off-target sites were predicted on the website http://crispr.hzau.edu.cn/CRISPR/. DNA fragments containing the off-target sites were amplified by PCR using KOD DNA polymerase (Aidlab, Beijing, China). Then, the PCR products were sequenced and analyzed for detecting the off-target events. All the primers are in Additional file 1: Table S1.

### Trait Measurement

All the transgenic and control plants were grown in the field under natural conditions with a plot size 100 × 180 cm^2^. The plant height, panicle length, panicle number, panicle branch number and grain number per panicle were obtained at the mature stage. The grain length, width, and thickness were measured by an electronic digital display Vernier caliper and fully filled grains were used for determined the 1000-grain weight. For grain quality, chalky percentage and degree of chalkiness were measured as the previous described (Hu et al. 2015b).

### Subcellular Location of the OsSPL4

To verify the subcellular localization of OsSPL4 protein, the complete coding sequence without stop codon was amplified and cloned into the HBT-GFP vector driven by the cauliflower mosaic virus 35S promoter. The 35S:OsSPL4-GFP fusion vector and nuclear localization vector (NLS-mCherry) were co-conducted into protoplast via PEG4000 inducing. After incubated in 28 °C for 16 h–20 h, the green and red fluorescence were observed with a confocal laser scanning microscope.

### Scanning Electron Microscopy

Fresh young panicles were harvested and immediately fixed in 2.5% glutaraldehyde (in 25 mM phosphate buffer, pH 7.0) overnight and then dehydrated through an ethanol series. Then, the samples were dried to critical point and sputter-coated samples were observed using a scanning electron microscopy (SEM) (Hitachi S-3400 N, Japan) as described previously (Gao et al. 2015).

### Dual Luciferase (LUC) Analysis in Tobacco Leaves

The reporter gene constructs (GAL4-UAS) and effector constructs (SPL4, SPL4-d3, SPL4-d15) were performed as previously described (Lyu et al., 2020). Briefly, the GAL4-UAS was cloned and inserted into the pGreenII 0800-LUC vector as the reporter. The full-length CDSs of OsSPL4, OsSPL4-d3, OsSPL4-d15 were inserted into the pGreenII 62-SK vector as effectors. The empty vector pGreenII 62-SK was used as a negative control. The *Agrobacterium* strain containing both the reporter pGreenII0800-LUC and the helper pSoup-P19 was used either alone or mixed with the *Agrobacterium* strain containing the effector plasmids. Overnight cultures of *Agrobacterium* were collected by centrifugation resuspended and infiltrated. After three days, *N. benthamiana* leaf samples were collected for the Dual-LUC assay using commercial Dual-LUC reaction (DLR) reagents (Promega, USA) according to the manufacturer’s protocol. The ratio of LUC to REN activities was calculated as an indication of the final transcriptional activity. Three biological repeats were measured for each sample.

### RNA Ligase-Mediated 5′-RACE

Total RNA from rice young panicles was ligated directly to the 5′RACE adapter using the 5′-Full RACE Kit (Takara, Japan) according to the manufacturer’s protocol as described (Yi et al. 2013). The first and second rounds of PCRs were performed with the *OsSPL4*-specific primers GSP1 and GSP2 (Additional file 1: Table S1). After PCR amplification, the PCR products were gel-purified and cloned into pMD18-T vector (Takara) for sequencing.

### RNA-Sequencing and Co-expression Network Construction

Total RNA was extracted from young panicles (P2 ~ P4) and flag leaves of the control plants and *spl4-d3* transgenic lines. Libraries were constructed and sequenced using an Illumina HiSeq 4000 platform with three biological replicates. After filtering, the clean reads were performed using the TopHat and Cufflinks package (version 2.2.1) (Trapnell et al. 2012). And DEGs were identified using DESeq2 with false discovery rate (FDR) < 0.05 (Love et al. 2014). Gene ontology (GO) and KEGG enrichment analysis were performed using AgriGO and KOBAS3.0 (http://kobas.cbi.pku.edu.cn/) with *P* < 0.05, respectively (Du et al. 2010; Xie et al. 2011). The heatmaps of differential expression patterns were performed by MeV4.7 (Saeed et al., 2003). Gene regulatory interactions and gene networks were download from RiceNet v2 (https://www.inetbio.org/ricenet/) (Lee et al. 2015). Then, co-expression networks were visualized by Cytoscape 3.6.1 (Shannon et al. 2003). All the clean reads were deposited in the National Center for Biotechnology Information (NCBI) under the accessions: PRJNA773224.

### Quantitative Real Time RT-PCR

Total RNA was extracted using TRIzol reagent (Invitrogen) from leaves, young panicles or other tissues in the transgenic plants and control plants. RNase-free DNase was used to degrade contaminated DNA from total RNA at 37 °C for 30 min. For mRNA reverse transcription (RT-PCR), the first strand cDNA was synthesized from 5 μg total RNA with Oligo(dT) primer according to the manufacturer’s instructions (Thermofisher Scientific). For miRNA RT-PCR, 2 μg of total RNA was reverse transcribed using miRNA-specific stem-loop primers (Varkonyi-Gasic et al. 2007). The reactions were incubated for 30 min at 16 °C, followed by 60 cycles of 30 °C for 30 s, 42 °C for 30 s and 50 °C for 1 s, then terminated by heating at 70 °C for 5 min. All the primers are listed in Additional file 1: Table S1.

The qRT-PCRs were carried out with a StepOnePlus Real-Time PCR System (Applied Biosystems). The *β-actin* gene and U6 snRNA were used as an internal control for mRNA and miRNA qRT-PCR analysis, respectively. All cDNAs were diluted 5 times and 1 μl diluted product was mixed with 5 μl of 2 × SYBR Green mix (Roche) and 0.25 μM primers in a 10 μl volume reaction system, which were incubated 10 min at 95 °C, followed by 40 cycles of 1 min at 60 °C. Three replicates were carried out for each sample, and the melting curve was performed to avoid nonspecific amplification. The relative expression levels were calculated using ^△△^ Ct method. All primers used in the present study are listed in Additional file 1: Table S1.

### Gene Structure and Protein Structure Analysis

The exon and intron structures of individual *OsSPL* genes were illustrated using the GSDS website (Gene Structure Display Server, http://gsds.cbi.pku.edu.cn/) by aligning the genomic DNA sequences and the corresponding transcript sequences from the RAP-DB or RGAP databases (Hu et al. 2015a). Homology modelling of OsSPL4 protein was carried out using Swiss-Pdb View 4.1.0 with the *Arabidopsis* SPL4 (PDB: 1ul4) as the template. Protein structure and surface were visualized using PyMol software (https://pymol.org/).

### Phylogenetic Analysis of the *SPL* Genes

Multiple sequence alignments of the 19 *OsSPL* gene and protein sequences were conducted using ClustalX software, and phylogenetic trees of the genes and proteins in rice were constructed with MEGA 7.0 software, respectively (Kumar et al. 2016). The phylogenetic trees were constructed using a neighbor-joining (NJ) method with bootstrap analysis of 1000 replicates.

### Nucleotide Diversity and Haplotype Network Analysis

Nucleotide diversity (π) was calculated to investigate the domestication of *OsSPL4* in rice using 10 wild and 3010 cultivated rice accessions (Zheng et al. 2015). Fixation statistics (*F*_ST_) among different subpopulations was calculated with sliding windows of 20 kb across the chromosome 2 based on the genetic variations of 529 rice accessions (Zhao et al. 2015). Haplotype network was constructed using an R package pegas with some modifications, and only haplotypes detected in more than 10 rice accessions were used.

## Results

### Mutation of *OsSPL4* Has Pleiotropic Effects on Rice Development

**Fig. 1:**
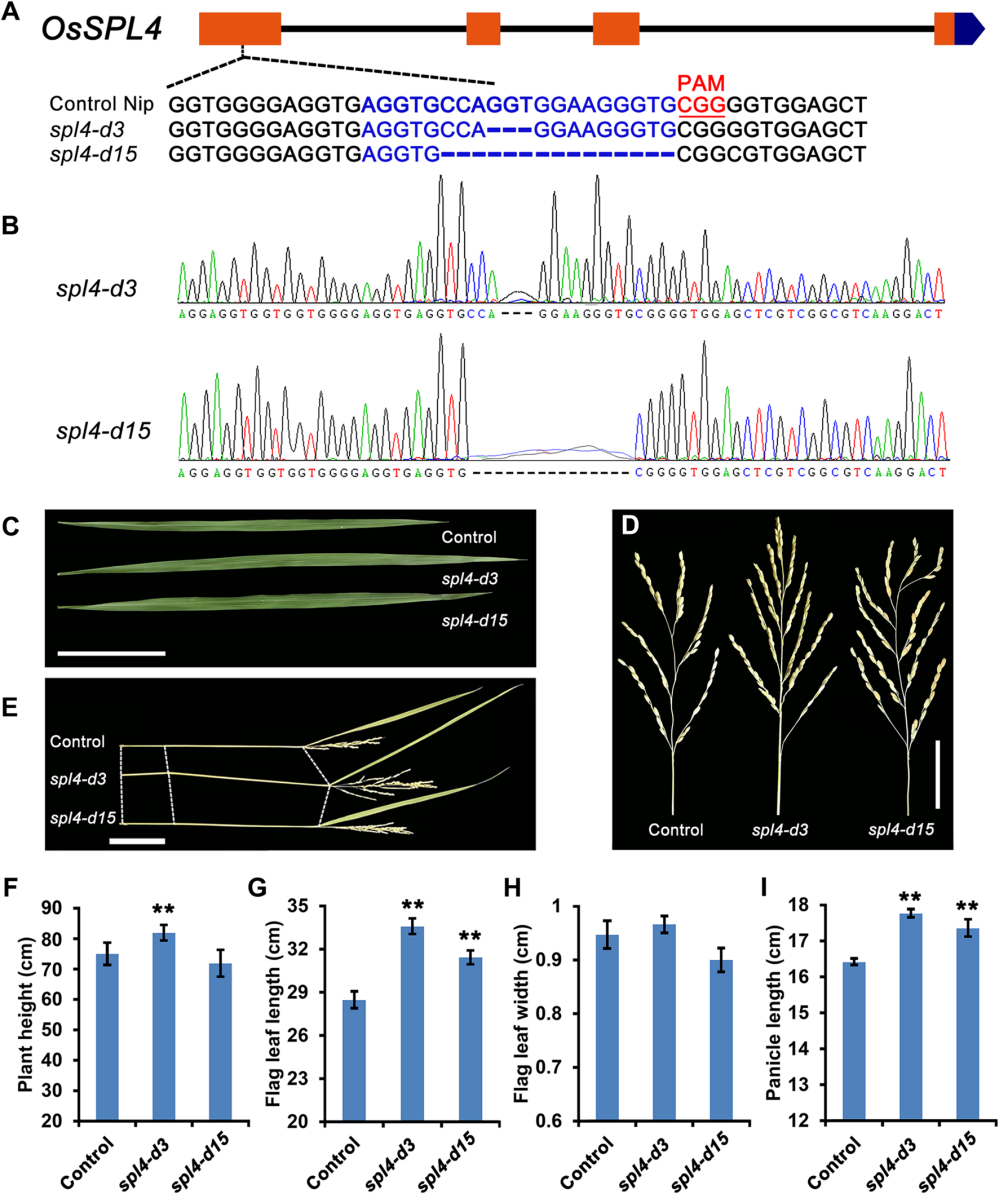
Pleiotropic effects of the mutants of *OsSPL4* in Nipponbare background. **A** Gene structure of *OsSPL4* and the sequence alignment between the mutants in T3 generation and control plants. Nip, Nipponbare. The deleted amino acid residues in the mutants are presented by dashes. The protospacer adjacent motif (PAM) are underlined in red. Two types of mutation events (*spl4-d3* and *spl4-d15*) were generated by CRISPR/Cas9 in Nipponbare rice. **B** The two different mutation events were confirmed by Sanger sequencing. **C**–**H** Comparisons of several important traits among *spl4-d3*, *spl4-d15* lines and control plants. **C** Flag leaf, scale bar = 10 cm. **D** Panicle, scale bar = 5 cm. **E** Stem, scale bar = 10 cm. **F** Plant height. **G** Flag leaf length. **H** Flag leaf width. **I** Panicle length. *P* values were determined by Student’s *t* test. *P < 0.05, **P < 0.01

To get a better insight into the role of SBP domain transcription factors in plant development, a CRISPR/Cas9 system was used to generate mutants of the SBP-box genes. In the present study, we identified the mutation lines of *OsSPL4* to affect panicle development and grain size (Fig. 1). The CRISPR/Cas9 constructs targeted the SBP domain in the first exon of *OsSPL4* was generated, which yielded 3 bp (*spl4-d3*) and 15 bp (*spl4-d15*) deletion, respectively (Fig. 1A, B). The potential off-target events in the mutation lines were also examined and the results showed that two of the six sites experience off-target events (10% ~ 20%) (Table 1). Interestingly, all these *OsSPL4* mutation lines showed the similar phenotypes and might influence the plant height in the field experiments (Fig. 1C-I; Additional file 1: Fig. S1). These mutation lines exhibited long flag leaf, increased panicle length with more spikelets per panicle, and even increased the grain size (Fig. 1C-F). However, tiller number per plant of the mutation lines in the field experiments showed no significant difference (Additional file 1: Fig. S1). In addition, for grain quality, the chalky percentage and degree of chalkiness were increased in the mutation lines (Additional file 1: Table S2). Therefore, the results suggested that *OsSPL4* plays an important role in panicle and grain development.

**Table 1:** The putative off-target event in the *osspl4* mutant lines

Target site	Putative off-target site	Sequence of putative off-target site	Region	No. of plants	No. of plants with mutations	Mutation rate (%)
*OsSPL4*-OFF1	Chr7:19100187-19100209	AGGTGCCAGGTGGA*GA*GGTG**CGG**	CDS	10	2	20%
*OsSPL4*-OFF2	Chr8:25274878-25274900	*C*GGTGCCAGGTGGA*G*GGGTG**CGG**	CDS	10	1	10%
*OsSPL4*-OFF3	Chr9:18918476-18918498	AGGTGCCAGGTGGA*G*GG*T*TG**CGG**	CDS	10	0	0
*OsSPL4*-OFF4	Chr8:167437-167459	*T*GGTGC*T*AGG*A*GGAAGGGT*T***TGG**	Intergenic	10	0	0
*OsSPL4*-OFF5	Chr9:16055179-16055201	AG*AG*GCC*T*GGTGGAA*A*GGTG**AGG**	Intron	10	0	0
*OsSPL4*-OFF6	Chr1:18039404-18039426	A*A*G*C*GCCA*T*GTGGA*G*GGGTG**TGG**	Intergenic	10	0	0

The bold font represents the PAM motif (NGG), the italics font indicates the mismatch bases

### Mutation of *OsSPL4* Increases Grain Weight

**Fig. 2:**
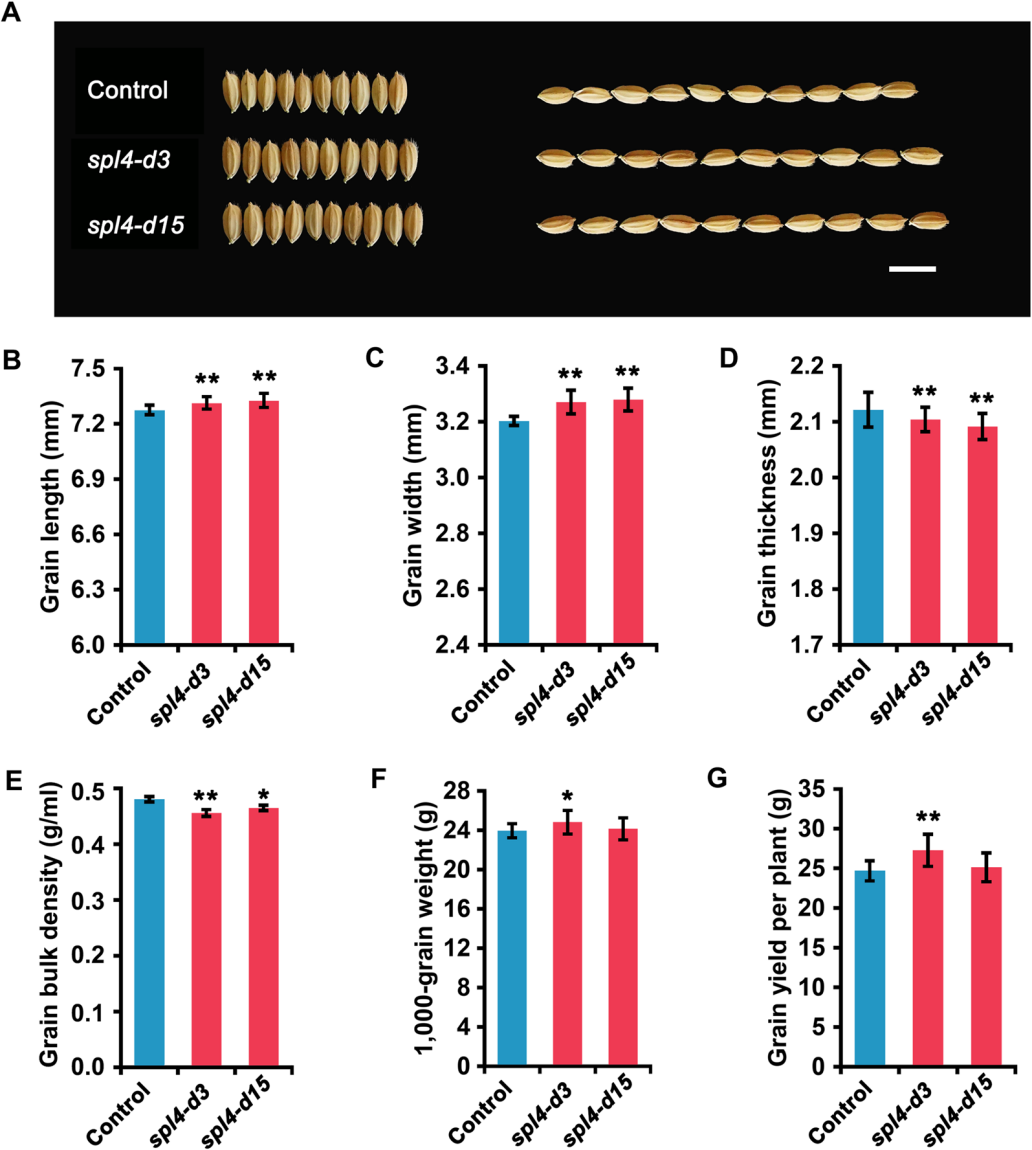
Grain phenotypes of the *OsSPL4* mutation transgenic lines and control plants. **A** Grain shapes of *spl4-d3*, *spl4-d15* and control plants, scale bar = 1 cm. **B**–**G** Comparisons of grain size and the yield between *spl4-d3*, *spl4-d15* and control plants. **B** Grain length. **C** Grain width. **D** Grain thickness. **E** Grain bulk density. **F** 1000-grain weight. **G** Grain yield per plant. Significant difference was determined by Student’s *t* test, *P < 0.05, **P < 0.01

The spikelet hull of *OsSPL4* mutation transgenic plants was apparently larger than that of control plants both in length and width (Fig. 2A). The grain length of *spl4-d3* and *spl4-d15* lines was significantly longer than that of the control plants, and grain width were also increased in *spl4-d3* and *spl4-d15* mutation lines, while the grain thickness were decreased (Fig. 2B-D). And the grain bulk density in *spl4-d3* and *spl4-d15* lines were significantly decreased (Fig. 2E). Thus, the 1000-grain weight was increased in *spl4-d3* and *spl4-d15* lines, compared with the control plants. Furthermore, the grain yield per plant was significantly increased by 11.44%, and the yield per plot was also increased by 5.35% in the *spl4-d3* lines, but the yield in *spl4-d15* lines were not significantly increased (Fig. 2F, G and Additional file 1: Table S2).

### Overexpression of *OsSPL4* Decreases Grain Weight

**Fig. 3:**
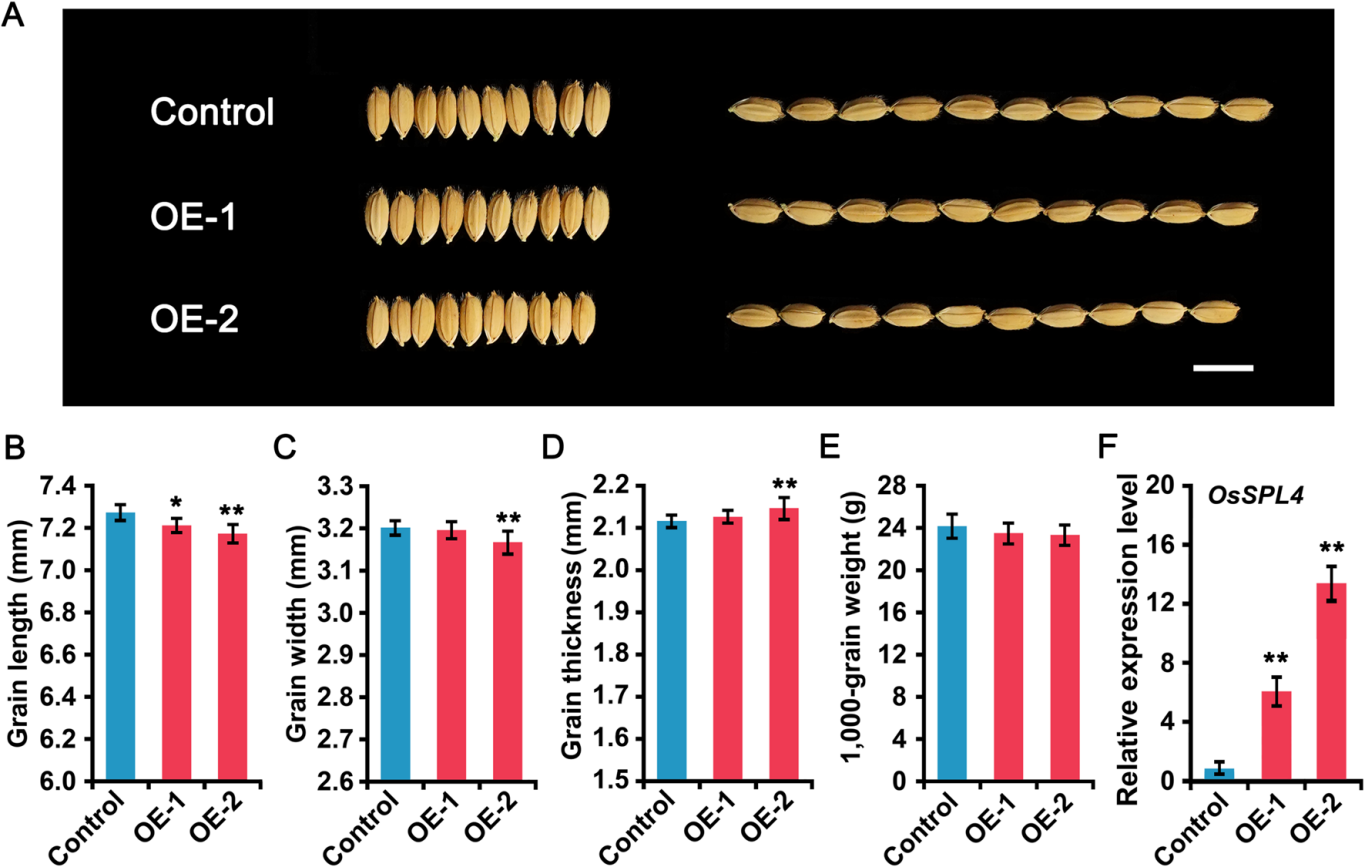
Overexpression of *OsSPL4* decreases rice grain size and weight. **A** Grain comparisons of the control and *OsSPL4* overexpression plants, scale bar = 10 mm. **B**–**E** Comparisons of the grain size and expression patterns in the control and *OsSPL4* overexpression plants. **B** Grain length. **C** Grain width. **D** Grain thickness. **E** 1000-grain weight. **F** The expression levels of *OsSPL4*. Significant difference was determined by Student’s *t* test, *P < 0.05, **P < 0.01

To further confirm the role of *OsSPL4* in controlling grain size, we generated an overexpression construct driven by the Ubi promoter and introduced it into Nipponbare rice. Compared with the control plants, the grain size and grain weight in overexpression lines were decreased (Fig. 3). One of the transgenic lines (OE-2) showed apparently decrease in grain length and grain width as well as 1000-grain weight, while the grain thickness were increased (Fig. 3A-E). And the decreased grain size was further confirmed to be the consequence of highly expression level of *OsSPL4* with 6.0 and 13.5 fold increase in OE-1 and OE-2 lines, respectively (Fig. 3F). These results further suggested that *OsSPL4* regulated grain size in rice.

### Mutation of *OsSPL4* Increases Grain Size by Promoting Cell Division

**Fig. 4:**
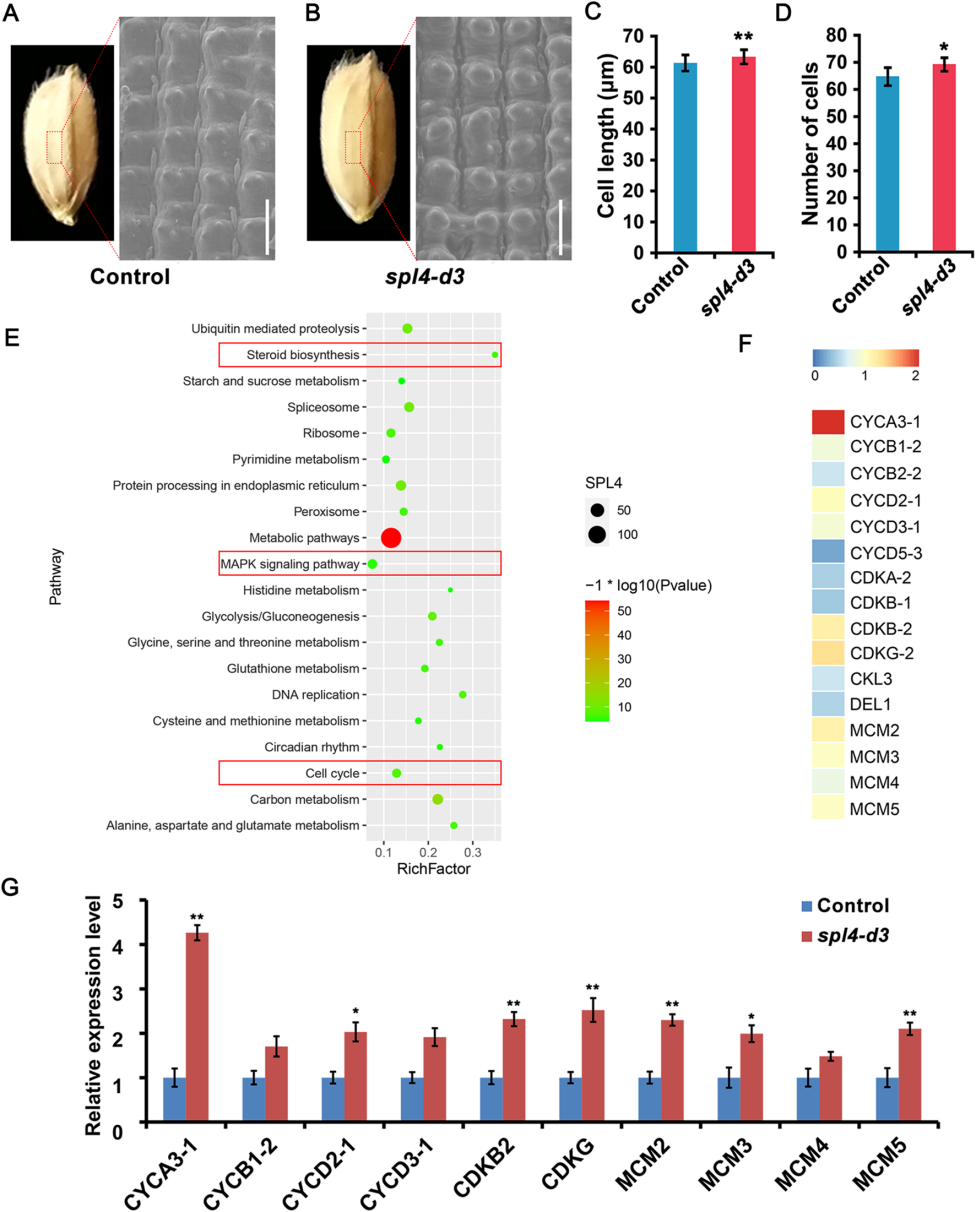
*OsSPL4* regulates spikelet hull development by affecting cell proliferation **A**, **B** Scanning electron microscope images of the outer glume (middle), **A** Control plants; **B** *spl4-d3* lines, scale bar = 100 μm. **C**, **D** Comparisons of cell length and cell number in the longitudinal direction of the spikelet between the control and *spl4-d3* plants (n = 10 spikelets). **C** Cell length. **D** Cell number. **E** KEGG pathway of the DEGs identified in young panicles of the control and *spl4-d3* transgenic plants. RichFactor is the ratio of DEG number and background gene number. **F** Heatmap showing the expression levels of genes related to floral organ identify in our RNA-seq between the *spl4-d3* lines and control plants. Fold change of the FPKM values (*spl4-d3*/control) were used. **G** Comparisons of the expression levels of cell-cycle genes in young panicles of the control and *spl4-d3* plants using qRT-PCR. The data are presented as mean ± SD (n = 3). Significant difference was determined by Student’s *t* test, * *P* < 0.05, ** *P* < 0.01

Compared with the *spl4-d15* lines, the 3 bp mutation of *OsSPL4* ( *spl4-d3*) transgenic plants exhibited better field phenotypes, which could be an excellent allele for regulating grain size in rice (Figs. 1, 2 and Additional file 1: Table S2). Therefore, we used the *spl4-d3* lines for further functional studies. Scanning electron microscopy of the outer glume revealed that *spl4-d3* lines exhibited a significantly enlarged cell volume than that of control plants (Fig. 4A, B). And the cell length in longitudinal direction was larger in *spl4-d3* lines with an increase in epidermal cell numbers per unit area (6.85%) (Fig. 4C, D). Furthermore, young panicles of the *spl4-d3* transgenic lines and control plants were analyzed using RNA-seq. KEGG pathway analysis revealed that many differentially expressed genes (DEGs) were involved in cell cycle pathway, MAPK signaling pathway and steroid biosynthesis (Fig. 4E). Many genes in the cell cycle pathway were highly expressed in the *spl4-d3* transgenic plants (Fig. 4F and Additional file 1: Table S3). The expression levels of 10 cell-cycle related genes were validated by qRT–PCR, and many of these genes, including *CYCA3-1* and *CYCD2-1* were significantly up-regulated (Fig. 4G). These results suggested that a higher expression of the cell-cycle genes probably promoted cell proliferation, contributing to affect grain size in *spl4-d3* mutation transgenic plants, which might potentially be useful for the improvement in rice yield.

### Mutation of *OsSPL4* Promotes Panicle Branching

**Fig. 5:**
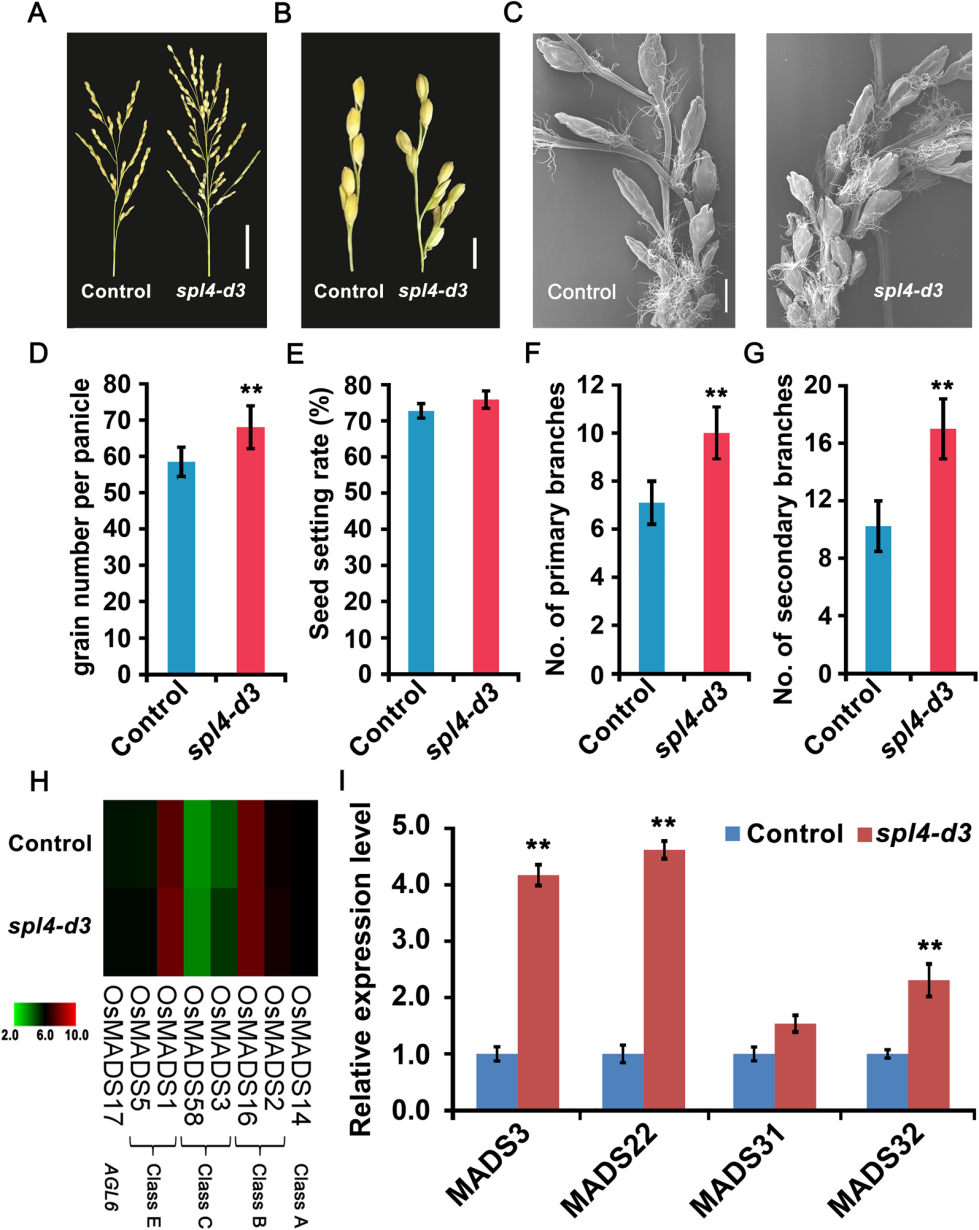
Comparisons of the panicle morphologies of *spl4-d3* transgenic plants with control plants. **A** Panicle architecture, scale bar = 5 cm. **B** Secondary branch, scale bar = 1 cm. **C** Scanning electron micrographs of the young panicle, scale bar = 4 mm. **D** Grain number per panicle. **E** Seed setting rate. **F** Number of primary branches. **G** Number of secondary branches. **H** Heatmap of the expression patterns of the floral identity genes. **I** qRT-PCRs validation of the expression levels of some flower development genes (*MADS3*, *MADS22*, *MADS31* and *MADS32*) in *spl4-d3* transgenic plants. Data were normalized to those of the control plants. Values are means ± SD (n = 3). Significant difference was determined by Student’s *t* test, *P < 0.05, **P < 0.01

In our study, *spl4-d3* transgenic plants possessed more spikelets per panicle and effective grains per panicle than those of control plants and *spl4-d15* lines, resulting in more grain number and significantly increase in rice yield (Fig. 5A-D, Additional file 1: Table S2). The seed setting rate was also slightly increased in the *spl4-d3* lines (Fig. 5E). The increased spikelets of *spl4-d3* lines were attributed to the increase of both primary branches and secondary branches (Fig. 5B, F, G). Scanning electron microscopy also showed that much more spikelets in the *spl4-d3* lines than those of control plants (Fig. 5C). Using RNA-seq, many floral identity genes in classes A, B, C, E, and *AGL6* of the ABCDE model were observed to be up-regulated in the young panicles (Fig. 5H, Additional file 1: Table S3). Further qRT-PCRs validated the highly expression levels of some MADS genes in the *spl4-d3* lines (Fig. 5I). The results indicated that *OsSPL4* regulated some of the floral genes in the mutation lines, which may eventually result in more panicle branches and grains per panicle. The number of panicle branches determines the number of grains, which is a major factor in affecting grain yield in rice.

### Identification of Genes Regulated by *OsSPL4*

To further investigate the molecular mechanism of *OsSPL4* on panicle and flag leaf development as well as affecting grain size, RNA-seq analysis was carried out using the young panicles at P2 ~ P4 stages and flag leaves from the control and *spl4-d3* transgenic plants, respectively. A total of 3,816 and 238 DEGs were identified in the young panicles and flag leaves, respectively (Additional file 1: Fig. S2A). GO analysis showed that “metabolic process”, “macromolecular complex assembly” and “oxidation reduction” were significantly enriched in the tissues between control and *spl4-d3* lines (Additional file 1: Fig. S2B,C). KEGG pathway analysis revealed that many DEGs participated in carbon metabolism, MAPK signaling, amino acid metabolism and ubiquitin mediated proteolysis (Fig. 4E; Additional file 1: Table S4). Particularly, we detected 16 and 7 DEGs to be involved in cell cycle and steroid biosynthesis, respectively. And a total of 21 MADS-box genes and 18 cell-cycle genes were up-regulated in the *spl4-d3* lines (Fig. 5H, Additional file 1: Table S3). Quantitative RT-PCR also validated the expression patterns of the four *OsMADS* genes and 10 cell-cycle genes (Fig. 4G, Fig. 5I).

To date, many rice yield-related genes, such as *RFL*, *OsGIF1*, *RGG2*, *DEP2*, *LAC*, *TGW3*, and *GL7/SLG7* have been reported to play essential roles in controlling the panicle architecture and grain size (Hu et al. 2020; Miao et al. 2019). In our study, we found that many of these yield-related genes had been differentially expressed in the *spl4-d3* lines (Additional file 1: Table S5). Especially, several genes including *GL3.3/TGW3* (*OsSK41*), *LAC*, and *RGG2* which negatively regulated rice grain size, were significantly down-regulated in the *spl4-d3* lines. By contrast, other genes of *OsGIF1*, *RGB1*, and *GL7/SLG7* were highly expressed in the *spl4-d3* lines (Additional file 1: Table S5). In addition, some of the BR signaling genes, including *BIM2* and *OsBAK1* were also slightly up-regulated in the *spl4-d3* lines (Additional file 1: Table S4). Co-expression network revealed that the regulation network of *OsSPL4* interacted with the yield-related genes, indicating the possible pathways of *OsSPL4* contributed to rice yield (Additional file 1: Fig. S3).

### The Role of SBP Domain in OsSPL4 Protein

There are 19 *OsSPL* genes in the rice genome, which have 3 to 11 exons (Additional file 1: Fig. S4A). Sequence analysis of the 19 *OsSPL* genes showed that *OsSPL4* has synteny with *OsSPL11* on chromosome 6 for segmental duplication in rice (Additional file 1: Fig. S4A). However, the last exon of *OsSPL4* is degenerated to the 3′-UTR which retained the OsmiR156 target site. Phylogenetic analysis of the OsSPL proteins revealed that the rice OsSPLs could be divided into 6 subgroups based on their different domains (Additional file 1: Fig. S4B). All of the rice OsSPL proteins have SBP domain. Expectedly, the syntenic OsSPL proteins were clustered in the same group and had the similar protein structures (Additional file 1: Fig. S4B).

**Fig. 6:**
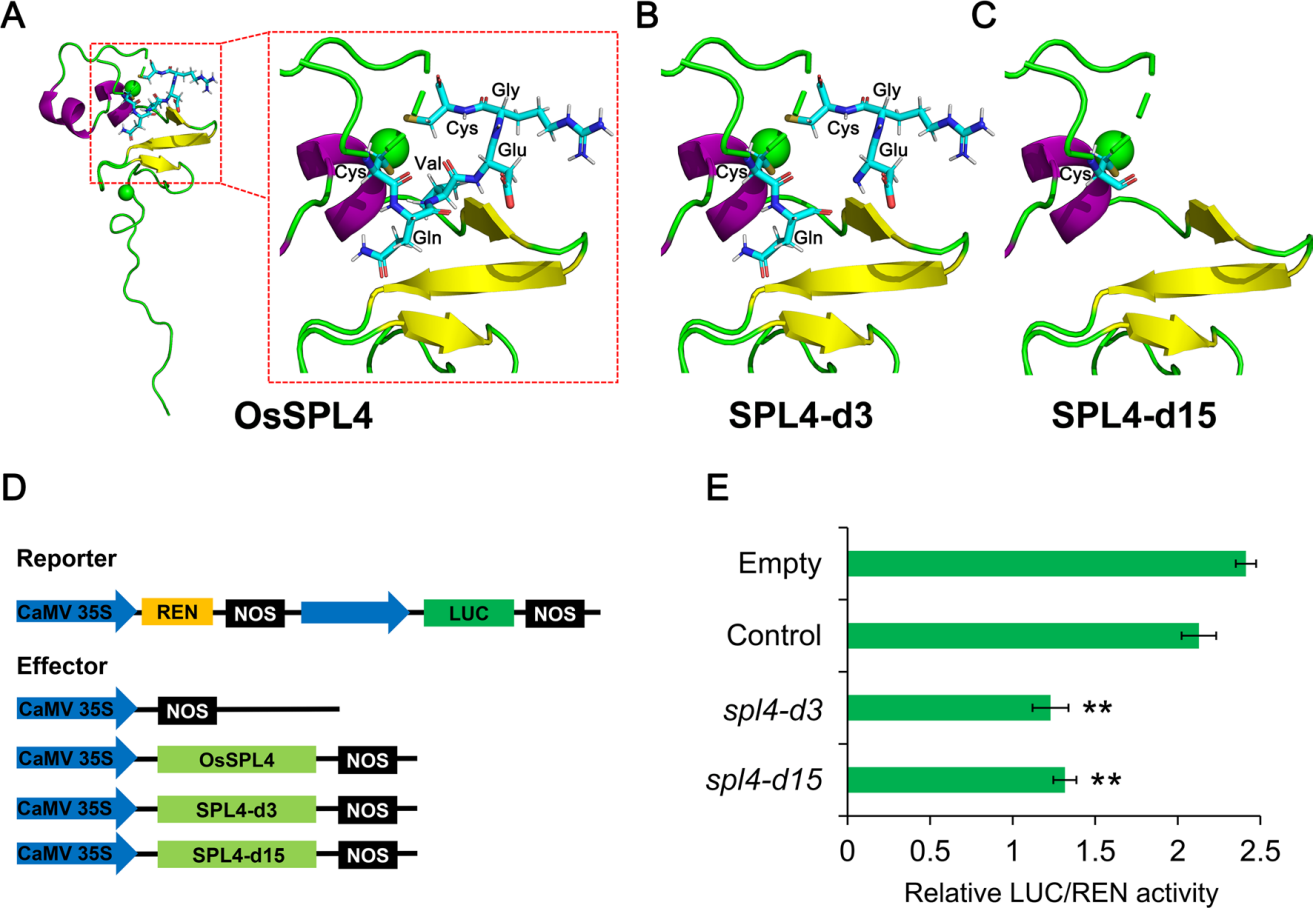
Three-dimensional (3D) structures of the control and *spl4-d3* plants and expression patterns of OsSPL4. **A** Three-dimensional homology modeling of the OsSPL4 protein based on crystallography data from *Arabidopsis* AtSPL4 structure (PDB ID: 1ul4.pdb). **B** The 3D structure of OsSPL4 in *spl4-d3* lines. **C** The 3D structure of OsSPL4 in *spl4*-*d15* lines. **D** Schematic diagram of the reporter and effector constructs for dual-luciferase transient assays. **E** Effects of OsSPL4 in *spl4-d3* and *spl4-d15* on the transcriptional regulation in *Nicotiana benthamiana*. Values are means ± SD (n = 3). Significant difference between control and *spl4-d3* or *spl4-d15* was determined by Student’s *t* test, * *P* < 0.05, ** *P* < 0.01

In plants, the SBP-DBD that consists of two subdomains has a single zinc-binding pocket for each one, which is likely to be stabilized through interactions of hydrophobic residues, such as Val, Phe and Tyr (Yamasaki et al. 2006). In our study, we found that OsSPL4 also contains the typical SBP domain with two nuclear location signals (NLSs) (Fig. 6A and Additional file 1: Fig. S5). And the mutations of *spl4-d3* and *spl4-d15* deleted one and five amino acids in the SBP domain, respectively (Additional file 1: Fig. S5). Particularly, one of the Cys residues which participates in the zinc binding was deleted in the *spl4-d15* mutation lines, resulting in not forming C3H subdomain. The 3D structure analysis also revealed that the lack of Val residues could affect formation of the first zinc-binding pocket in *spl4-d3* mutation of OsSPL4 (Fig. 6B). Whereas, the 15 bp mutation of OsSPL4 devastate the binding pocket due to the deletion of Cys residue (Fig. 6C).

To investigate whether the two mutation (SPL4-d3 and  SPL4-d15) of OsSPL4 were loss of functions, we performed a dual-luciferase transient transcriptional activity assay in *N. benthamiana* leaves with OsSPL4 driven by the CaMV 35S promoter as an effector and LUC as the reporter gene (Fig. 6D). *DEP1* is an important regulator for panicle architecture and elevated expression of *DEP1* contributed to increasing panicle length (Zhou et al., 2009; Lu et al., 2013). Previous studies have demonstrated that *OsSPL* genes could bind to the promoter of *DEP1* (Lu et al., 2013; Yuan et al. 2019). In our study, the results showed that the extent of DEP1::LUC were significantly reduced by cotransformation with *OsSPL4* in both SPL4-d3 and SPL4-d15 compared with the control plants (Fig. 6E), indicating the transcriptional activity of these two mutations of OsSPL4 were reducing. These results suggested that the two mutations of *OsSPL4* (*spl4-d3* and *spl4-d15*) in this study could affect the binding in promoter of regulated genes and then influence the regulation of downstream genes.

### Expression Pattern and Subcellular Location of *OsSPL4*

**Fig. 7:**
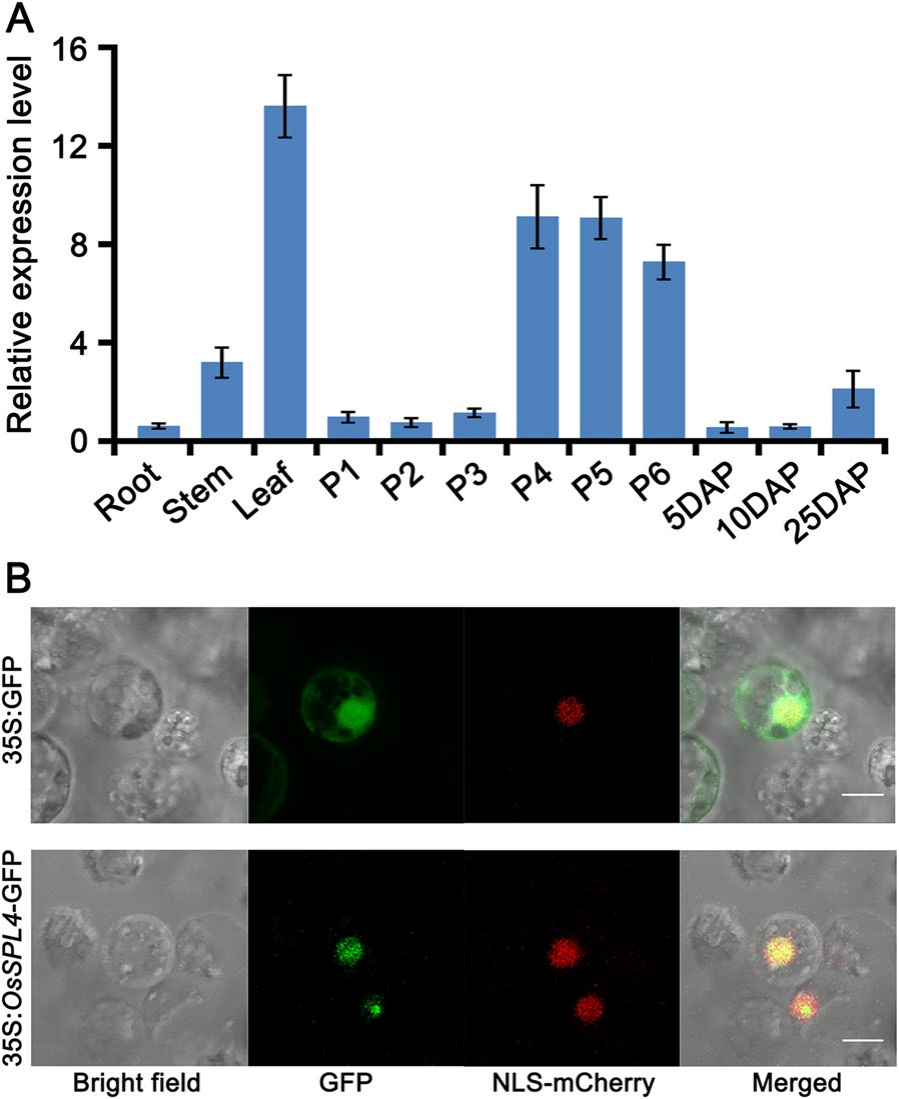
The expression patterns and subcellular localization of *OsSPL4*. **A** The expression patterns of *OsSPL4* in different tissues. P1–P6 indicates a plant with a panicle length of 0, 3, 5, 10, 15 and 22 cm, respectively. DAP, days after pollination. **B** Subcellular localization of OsSPL4-GFP fusion protein. Scale bar = 10 mm, applicable to all photos

To determine the tissue-specific expression levels of *OsSPL4*, qRT-PCR analysis in wild type plants revealed that the expression level of *OsSPL4* was highly detected in the leaf and young panicle at P4-P6 stage during panicle development (Fig. 7A). But the expression level of *OsSPL4* was weakly detected in roots, developing seeds of 5DAP and the 10DAP stages (Fig. 7A). Consistent with these expression patterns, mutation of *OsSPL4* showed that flag leaf length, panicle length and the number of primary panicle branches as well as grain size were changed (Fig. 1).

To determine the subcellular location, the coding sequence of *OsSPL4* was fused with GFP driven by the cauliflower mosaic virus 35S promoter. The results showed that green fluorescence from OsSPL4-GFP fusion proteins was coincident in the same position with red fluorescence from the nucleus marker vector NLS-mCherry, suggesting that OsSPL4 was located in the nucleus (Fig. 7B).

### *OsSPL4* is One of the Osa-miR156 Targets

Using psRNATarget, we predicted that *OsSPL4* can be targeted by many members of OsmiR156 (Additional file 1: Fig. S6A). And the target site of OsmiR156 is located in the 3′-untranslated region (UTR) of *OsSPL4*. A 5′RLM-RACE (RNA ligase-mediated rapid amplification of cDNA ends) analysis showed that osa-miR156 could directly cleave *OsSPL4* mRNA in vivo (Additional file 1: Fig. S6A). To further investigate whether the regulation of OsmiR156 on *OsSPL4* affects the grain size, we generated the overexpression and STTM constructs, and then they were introduced into Nipponbare rice (Additional file 1: Fig. S6B). Moreover, qRT-PCRs revealed that four of the eight predicted targets were up-regulated in the STTM156, while, only the *OsSPL4* significantly down-regulated in the OE-miR156 lines, indicating *OsSPL4* was a target of osa-miR156 (Additional file 1: Fig. S6C). The OE-miR156 transgenic plants exhibited a larger grain size, which increased the grain length and width (Additional file 1: Fig. S7). Pearson correlation analysis showed that the expression levels of *OsSPL4* in OE-miR156 lines  were significantly negatively correlated with grain length (*P* = 0.0286, R = 0.6285) (Additional file 1: Fig. S7C). Moreover, one of the rice STTM156 line (STTM156-4) showed that the grain length and width were obviously decreased (Additional file 1: Fig. S8). Consistent with the above results, *OsSPL4* transcript levels in the osa-miR156 overexpressing lines were lower than those in the control plants, whereas the expression level of *OsSPL4* increased in the STTM156 transgenic plants (Additional file 1: Fig. S7B and S8B). And the Pearson correlation analysis also revealed that the expression levels of *OsSPL4* in STTM156 lines  were significantly negative correlated with grain length (*P* = 0.0024, R = 0.7862) and grain width (*P* = 0.0031, R = 0.7748) (Additional file 1: Fig. S8E, F), respectively. These results suggested that *OsSPL4* was one of the targets of osa-miR156, and down-regulated *OsSPL4* by OsmiR156 might change the rice grain shape.

### Genetic Diversity of *OsSPL4* Gene

**Fig. 8:**
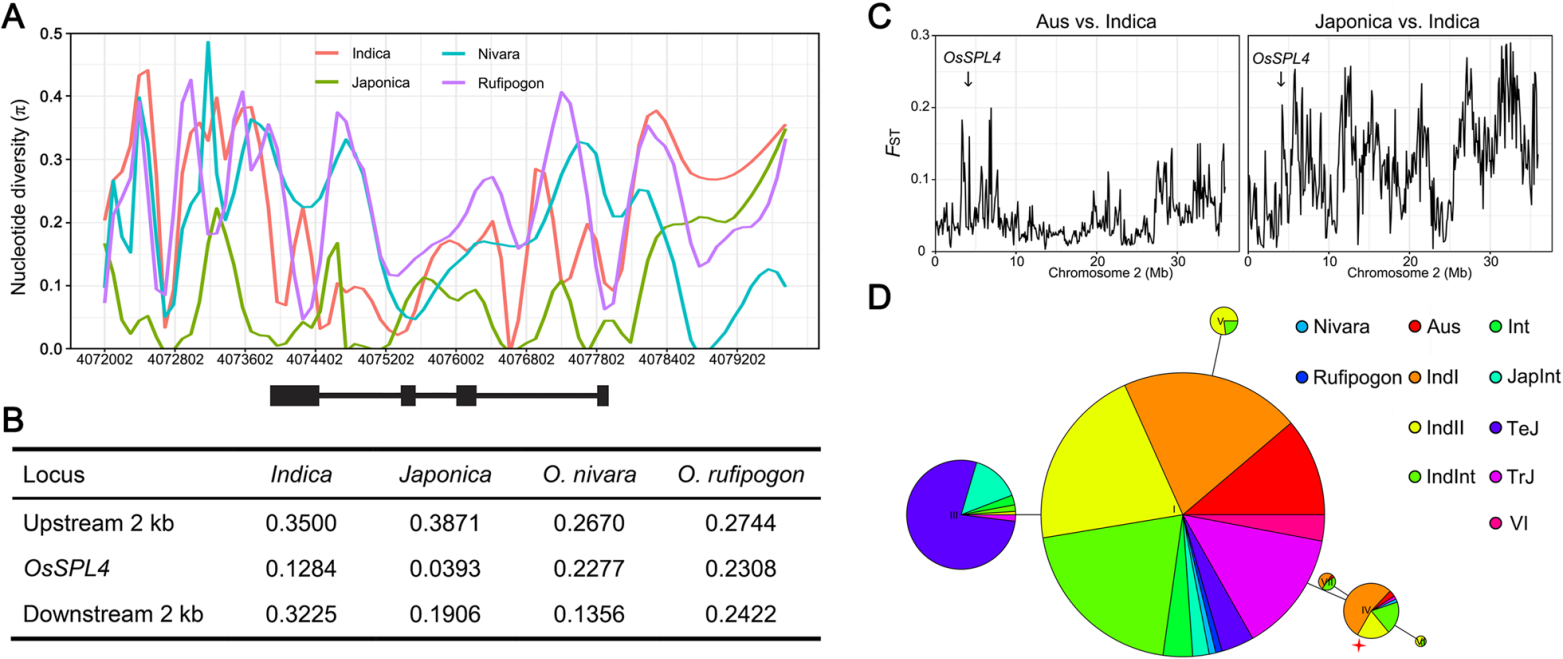
The nucleotide diversity and haplotype network analysis of *OsSPL4*. **A** Nucleotide diversity (π) analysis in *OsSPL4* gene and its flanking region (4 kb) based on the 10 wild and 3010 cultivated rice accessions. Nivara, *O. nivara*; Rufipogon, *O. rufipogon*. Y-axis indicates the π value and x-axis shows the genome location on chromosome 2 in rice. **B** Average nucleotide diversity (π) of *OsSPL4* and its flanking region (2 kb). **C** Genetic differentiation (*F*_ST_) across chromosome 2 between different subspecies ( *Aus* vs. *Indica* and *Japonica* vs. *Indica*). A total of 592 rice accessions were used for analysis, including 46 *aus*, 295 *indica* and 156 *japonica* rice from RiceVarMap database, respectively. **D** Haplotype network of *OsSPL4* among 10 wild and 592 cultivar rice accessions. Only haplotypes found in more than 10 rice accessions were used to construct the haplotype network. Each circle represents a haplotype and circle size is proportional to the haplotype frequency. Different colors refer to different rice subpopulations. Red star corresponds to *OsSPL4*^A92V^. IndI, *indica* I; IndII, *indica* II; IndInt, *indica* intermediate; Int, intermediate group between *indica* and *japonica* accessions; JapInt, *japonica* intermediate; Nivara, *O. nivara*; TrJ, tropical japonica; TeJ, temperate japonica; Rufipogon, *O. rufipogon*. VI, intermediate group between *indica* and *japonica*/*Aromatic*

Compared with the wild rice *SPLs*, we observed that last exon of *SPLs* (*OsSPL4* and *OglaSPL4*) in cultivated rice were truncated (Additional file 1: Fig. S9). The results indicated that *OsSPL4* may be domesticated from wild rice. To further determine whether *OsSPL4* has undergone artificial selection, we analyzed the genetic diversity in *OsSPL4* and its flanking regions (~ 4 kb) using 10 accessions of wild rice (5 accessions from *O. rufipogon* and 5 accessions from *O. nivara*) and 3010 cultivars of *O. sativa* (Fig. 8A). Compared with the nucleotide diversity (π) of wild rice *O. rufipogon* (π = 0.2308) and *O. nivara* (π = 0.2277), the nucleotide diversity in *OsSPL4* genome sequences was decreased in *indica* and *japonica* cultivars (π = 0.1284 and 0.0393, respectively) (Fig. 8B). The results revealed that the nucleotide diversity of *OsSPL4* gene in cultivars was lower than that in wild rice (*P* < 0.01), and that the diversity in *japonica* cultivars was lower than that in *indica* cultivars, suggesting *OsSPL4* might have undergone artificial selection. Moreover, we examined *OsSPL4* evolution by estimating the level of population difference (*F*_ST_) on chromosome 2 between different subpopulations. Pairwise measurements of *F*_ST_ showed that *F*_ST_ levels were truly higher between *indica* and *japonica* in the *OsSPL4* gene. However, markedly weaker differences were found between *aus* and *indica* in the *OsSPL4* when compared with the mean *F*_ST_ over chromosome 2 as a whole (Fig. 8C). A minimum-spanning tree of haplotypes based on the variation from RiceVarMap database in 529 rice accessions revealed two mainly distinct clusters: separate *indica* and *japonica* haplotype clusters (Fig. 8D). Furthermore, we also performed haplotype network analysis based on the Rice Functional Genomics and Breeding (RFGB) database, obtaining eight haplotypes for *OsSPL4* (Additional file 1: Fig. S10A). One of the missense mutation SNP (C-T) at Chr02:4,074,278 (vg0204074278) in Hap 4, 5, 8 could change the Ala into Val (A92V) in the SBP domain of OsSPL4, which was identified in both RiceVarMap and RFGB database (Additional file 1: Fig. S10A and Tables S6, S7). According to the phenotypes from RFGB and RiceVarMap database, the grain length of rice varieties in Hap 5 was longer than that of Hap 1 or Hap 2, indicating that the mutation of SBP domain in the OsSPL4 could affect the grain shape (Additional file 1: Fig. S10B and Table S8).

## Discussion

Although a number of grain yield-related QTLs/genes have been characterized in the past years, more yield genes are required for the high-yield rice breeding. SPL proteins are plant-specific transcription factors, which contain a SBP-box motif and participate in many crucial biological processes in plants (Guo et al. 2008). Although the biological functions and molecular mechanisms in many of the *OsSPL* family members have been extensively studied in rice, the underlying molecular mechanisms and regulation networks of *OsSPL4* were not very clear, especially in regulating the panicle branching and grain size (Jiang et al., 2020). In this study, we generated transgenic plants by mutating of *OsSPL4* using CRISPR/Cas9 system and overexpressing of *OsSPL4* to reveal its role in the regulation of panicle architecture and grain size in rice.

CRISPR/Cas9 which is a useful tool for targeted mutagenesis, can rapidly generate homozygous mutation transgenic plants in rice (Hu et al., 2020). Lately, CRISPR/Cas-mediated base editing systems have been developed for precise base editing with reducing indels and off-target changes in plants (Molla and Yang 2019). And using CRISPR/Cas9-mediated adenine base editors (ABEs), point mutation of *zebra3* and *wsl 5* in rice could affect the phenotypes (Molla and Yang 2019). It has been reported that two mutation types (3 bp and 39 bp deletion) of *OsIAA23* in Kasalath rice using the CRISPR/Cas9 system showed severe dwarfism, inhibited lateral root formation, reduced tiller number and exhibited lower seed-setting rate (Jiang et al. 2019). In rice, eight amino acid deletion in *RGG2* (*zrgg2-2*) and one amino acid deletion in *OsAOG17* (*ago17-2*) were also reported to regulate grain size and weight (Miao et al. 2019; Zhong et al. 2020). These results indicated that the deletion of amino acids could be resulted in alteration of agronomic traits. In the present study, two homozygous mutation (*spl4-d3* and *spl4-d15*) of *OsSPL4* with depressed activity could increase grain length and width as well as grain yield, although these deletions not cause any other changes in amino acid sequences (Fig. 3, Fig. 6E, Additional file 1: Fig. S1)*.*To further investigate the function of *OsSPL4* in rice, we successfully generated transgenic plants overexpressing *OsSPL4* in Nipponbare background. The OE1 and OE2 plants exhibited short panicles, decreased number of panicles and had small grains (Fig. 3; Additional file 1: Table S2). As one of the targets of OsmiR156, which targeted in the 3′ UTR region of *OsSPL4*, overexpression of osa-miR156 were found to increase the grain size in rice (Additional file 1: Figs. S6, S7). Whereas, the transgenic plants of STTM156 showed smaller grains (Additional file 1: Fig. S8).

The *spl4-d3* and *spl4-d15* transgenic plants, having amino acid mutations in the SBP domain of OsSPL4 protein, produced larger grains (Fig. 2). Since OsSPL4 is a plant-specific transcription factor, SBP domain is essential for binding of downstream target genes (Guo et al. 2008). Our three-dimensional structure analysis showed that the amino acids deletion of *spl4-d3* and *spl4-d3* affected the formation of zinc-binding pocket (Fig. 6A), indicating that the single valine deletion in *spl4-d3* might cause all effect probably by altering the binding properties of SBP domain to promoter of other downstream genes. *DEP1* has been reported to be directly bound with *OsSPL* genes, and elevated expression level of *DEP1* increased the panicle length (Lu et al. 2013; Yuan et al. 2019). In our study, the expression level of *DEP1* was up-regulated in the *spl4-d15* transgenic lines (Additional file 1: Table S5) and the transcriptional activity of *OsSPL4* were reduced in both *spl4-d3* and *spl4-d15* lines by the dual LUC assay (Fig. 6E).

Phylogenetic analysis reveals that *OsSPL4*, *OsSPL11*, *OsSPL3* and *OsSPL12* were grouped in the same subclade, in which *OsSPL11* was segmental duplication with *OsSPL4* (Additional file 1: Fig. S4) (Shao et al. 2019; Xie et al. 2006). Expression patterns analysis showed that *OsSPL4* was highly expressed in leaves, young panicles and developing seeds of 25DAP, indicating the roles in panicle development and grain size (Fig. 7A). Scanning electron microscopy showed that the out epidermal cells of the spikelet hulls in *spl4-d3* lines were much longer than that of control plants (Fig. 4A, B). And more grain number per panicle and panicle branches were observed in the *spl4-d3* lines (Fig. 5A-G). The field experiments also showed that the *spl4-d3* lines can increase the grain yield per plant by 11.44% in Nipponbare background (Fig. 2G, Additional file 1: Table S2). RNA-seq and qRT-PCRs validated that the floral identity genes and cell-cycle genes were highly expressed in the young panicles of *spl4-d3* lines (Fig. 4G, Fig. 5I, Additional file 1: Table S3). Hence, the *spl4-d3* transgenic plants had enlarged the spikelet hulls and increased grain number, which resulted in enhancing grain size and weight as well as yield in rice (Fig. 5). Although the grain quality of *OsSPL4* mutation lines have a little decrease, it could be improved by other approaches, such as crossing with high-quality sterile lines in the rice breeding (Hu et al. 2015b).

**Fig. 9:**
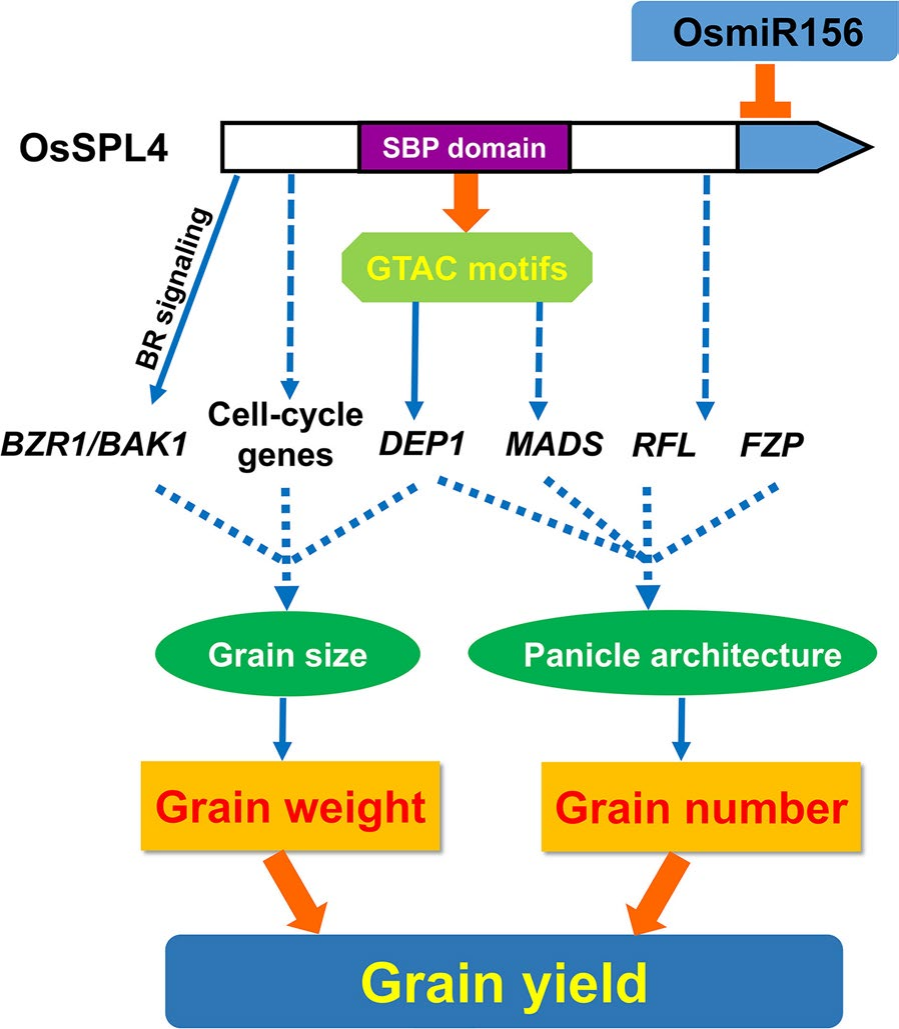
Aproposed model for the regulation of grain yield by OsSPL4 in rice. Arrows indicate the positive effects and short horizontal lines represent the negative effects. The dashed lines indicate the molecular mechanisms which are unclear. BR, brassinosteroid

We compared the expression patterns of several reported yield-related genes of rice between the control and *spl4-d3* transgenic plants. Co-expression network also revealed that *OsSPL4* was involved in the interaction networks of different yield-related genes (Additional file 1: Fig. S3). For example, *GW2* which negatively regulates grain width and weight, was down-regulated in *spl4-d3* lines (Additional file 1: Table S5) (Song et al. 2007). Three genes *LAX*, *FZP* and *RCN1* have been shown to control the axillary meristem initiation and development, which determined the panicle branching and grain number (Huang et al. 2009). Different expression patterns of these genes were found between the *spl4-d3* lines and control plants (Additional file 1: Table S5). Particularly, the expression level of *FZP* was repressed in *spl4-d3* lines, which has been reported to increase the number of spikelets per panicle and grain yield (Bai et al. 2017). Several yield-related genes were significantly up-regulated in *spl4-d3* transgenic plants, including *RFL*, *RGB1*, *GL7/GW7* and *OsGIF1* (Additional file 1: Table S5). In rice, *RFL* was reported to promote panicle branching by activating positive regulators such as *LAX* (Rao et al. 2008). *RGB1* positively regulates cellular proliferation and cell number (Utsunomiya et al. 2011). And the grain length and length of flag leaf were increased in the *GL7* overexpression lines (Wang et al. 2015). As the transcription coactivator of GRF, overexpression of *OsGIF1* has been reported to significantly increase grain size and weight (He et al. 2017; Li et al. 2016). Moreover, other yield-related genes such as *GL3.3/TGW3*, *RGG2* and *OsLAC*, were significantly suppressed in the *spl4-d3* transgenic plants (Additional file 1: Table S5). *GL3.3/TGW3* (*OsSK41*) and *RGG2* have been demonstrated to negatively regulate grain length and weight in rice (Hu et al. 2018a; Xia et al. 2018; Ying et al. 2016; Miao et al. 2019). *OsLAC* which is the target of OsmiR397b, is involved in the sensitivity of plants to BR. Overexpression of OsmiR397b repressed the expression of *OsLAC* and led to increase grain size and grain number per panicle (Zhang et al. 2013). Therefore, the mutation of *OsSPL4* in this study might regulate these yield-related genes or participate in BR signaling pathway to promote panicle branching and increase grain size, resulting in high yield in rice (Fig. 9).

Compared with the homologue *SPL4* genes in *Oryza* genus, the last exon of cultivated rice *SPLs* (*OsSPL4* and *OglaSPL4*) possessed a shorter length than other wild rice *SPLs* (Additional file 1: Fig. S9), which is consistent with the previous report (Zhong et al. 2019). The nucleotide diversity analysis of wild and cultivated rice population suggested that *OsSPL4* might have undergone artificial selection in the *japonica* rice (Fig. 6A). We identified a natural variation at Chr02:4,074,278 (C-T) of *OsSPL4* among both 3 K and 529 rice accessions (Additional file 1: Tables S6, S7). Further editing of *OsSPL4* at this site using the ABE system may effectively develop different grain length rice lines (Molla and Yang 2019; Molla et al., 2020). The A92V transition in the first exon, which is also located in the SBP domain, caused the change of amino acid from Ala to Val (Additional file 1: Fig. S10). This variant allele existed in the *aus* and *indica* group, which greatly contributed to grain length variance (Additional file 1: Fig. S10 and Additional file 2: Table S8). And population difference (*F*_ST_) was also showed to be lower between *aus* and *indica* than that of *indica* and *japonica* in the *OsSPL4* gene (Fig. 8C), indicating the allele in *indica* rice was introgressed from *aus* varieties under artificial selection. The results suggested that nonsense mutation occurred in the SBP domain could regulate the grain size and yield in rice.

## Conclusions

We identified two elite alleles of *OsSPL4*, which plays an important role in the panicle and grain development of rice. Mutation of *OsSPL4* in rice increased the number of grains per panicle and grain size based on the results of phenotyping, cytological observation, dual-luciferase assays, and RNA-seq analysis. And qRT-PCR also confirmed that several MADS-box and cell-cycle genes were shown to be regulated in the mutation lines. Hence, our findings not only identify *OsSPL4* as a key regulator of grain size by acting on cell division control but also suggest a strategy for grain size modification in a wide range of cereal crops for yield improvement.

## Supplementary Information

**Additional file 1: Fig. S1**. Plant phenotypes of overexpression (OE) and the mutant transgenic plants of OsSPL4. **Fig. S2**. Differentially expressed genes (DEGs) and GO enrichment in control and spl4-d3 plants using RNA-seq analysis. **Fig. S3**. Co-expression network of yield-related genes and OsSPL4 in rice. **Fig. S4**. Phylogenetic tree of the 19 SPL family members in rice. **Fig. S5**. Protein sequences and the structures in control and the mutation lines. **Fig. S6**. Schematic diagram of OsSPL4 as the target of OsmiR156 and the vector construction of STTM156. **Fig. S7**. Overexpresssion (OE) of osa-miR156 increases rice grain length and width. **Fig. S8**. Osa-miR156 mimicry (STTM156) transgenic rice decreases grain length and width. **Fig. S9**. Phylogenetic analysis and exon-intron structures of SPL4 orthologs in different Oryza genus. **Fig. S10**. Different haplotypes of OsSPL4. **Table S1**. Primers used in this study. **Table S2**. Grain yield and associated components in the transgenic rice in field. **Table S3**. Expression patterns of the floral and cell-cycle genes between control and spl4-d3 transgenic plants. **Table S4**. KEEG pathway of the differentially expressed genes in young panicle between spl4-d3 and control plants. **Table S5**. The expression patterns of rice yield-related genes between spl4-d3 and control plants. **Table S6**. Variations in OsSPL4 coding region identified from RFGB Database. **Table S7**. Variations in OsSPL4 coding region identified from RiceVarMap Database.

**Additional file 2: Table S8**. Phenotypes for the different haplotypes in OsSPL4 of rice.

## Data Availability

All the RNA-seq data were deposited in the National Center for Biotechnology Information (NCBI) under the accessions: PRJNA646823.

## Abbreviations

GO: Gene ontology
QTLs: Quantitative trait loci
DBD: DNA binding domain
FDR: False discovery rate
SPL: SQUAMOSA PROMOTER BINDING PROTEIN (SBP)-like
NCBI: National Center for Biotechnology Information
CRISPR/Cas9: Clustered regularly interspaced short palindromic repeats/CRISPR-associated protein-9
STTM: Short tandem target mimic
