# Supplementary material for: The Elite Alleles of OsSPL4 Regulate Grain Size and Increase Grain Yield in Rice
Source: Rice (N Y). 2021 Nov 2;14:90. doi: 10.1186/s12284-021-00531-7 (PMC8563897; doi:10.1186/s12284-021-00531-7)
Supplement: Supplementary file 1 — Additional file 1: Fig. S1. Plant phenotypes of overexpression (OE) and the mutant transgenic plants of OsSPL4. Fig. S2. Differentially expressed genes (DEGs) and GO enrichment in control and spl4-d3 plants using RNA-seq analysis. Fig. S3. Co-expression network of yield-related genes and OsSPL4 in rice. Fig. S4. Phylogenetic tree of the 19 SPL family members in rice. Fig. S5. Protein sequences and the structures in control and the mutation lines. Fig. S6. Schematic diagram of OsSPL4 as the target of OsmiR156 and the vector construction of STTM156. Fig. S7. Overexpresssion (OE) of osa-miR156 increases rice grain length and width. Fig. S8. Osa-miR156 mimicry (STTM156) transgenic rice decreases grain length and width. Fig. S9. Phylogenetic analysis and exon-intron structures of SPL4 orthologs in different Oryza genus. Fig. S10. Different haplotypes of OsSPL4. Table S1. Primers used in this study. Table S2. Grain yield and associated components in the transgenic rice in field. Table S3. Expression patterns of the floral and cell-cycle genes between control and spl4-d3 transgenic plants. Table S4. KEEG pathway of the differentially expressed genes in young panicle between spl4-d3 and control plants. Table S5. The expression patterns of rice yield-related genes between spl4-d3 and control plants. Table S6. Variations in OsSPL4 coding region identified from RFGB Database. Table S7. Variations in OsSPL4 coding region identified from RiceVarMap Database. [file 12284_2021_531_MOESM1_ESM.pdf]

Supplementary data

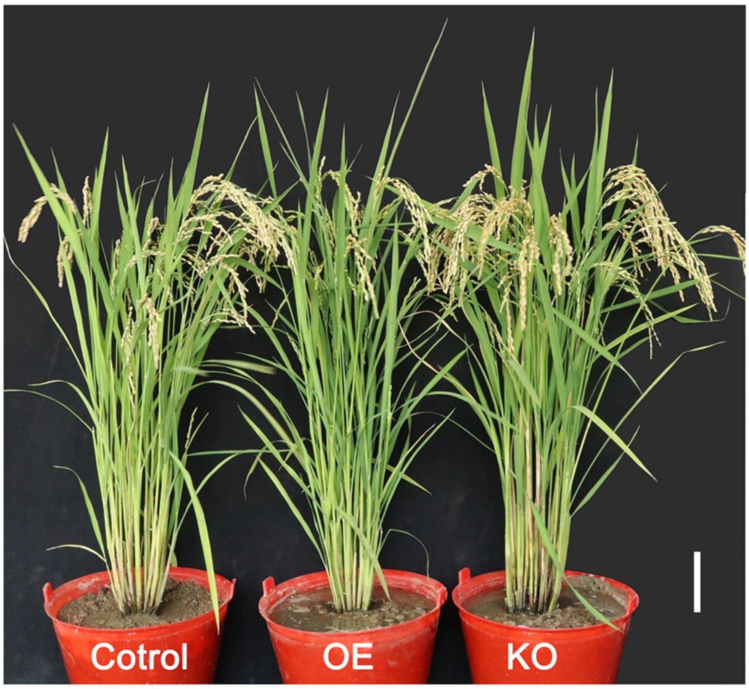

Figure S1. Plant phenotypes of overexpression (OE) and the mutant transgenic plants of *OsSPL4*. scale bar = 10 cm.

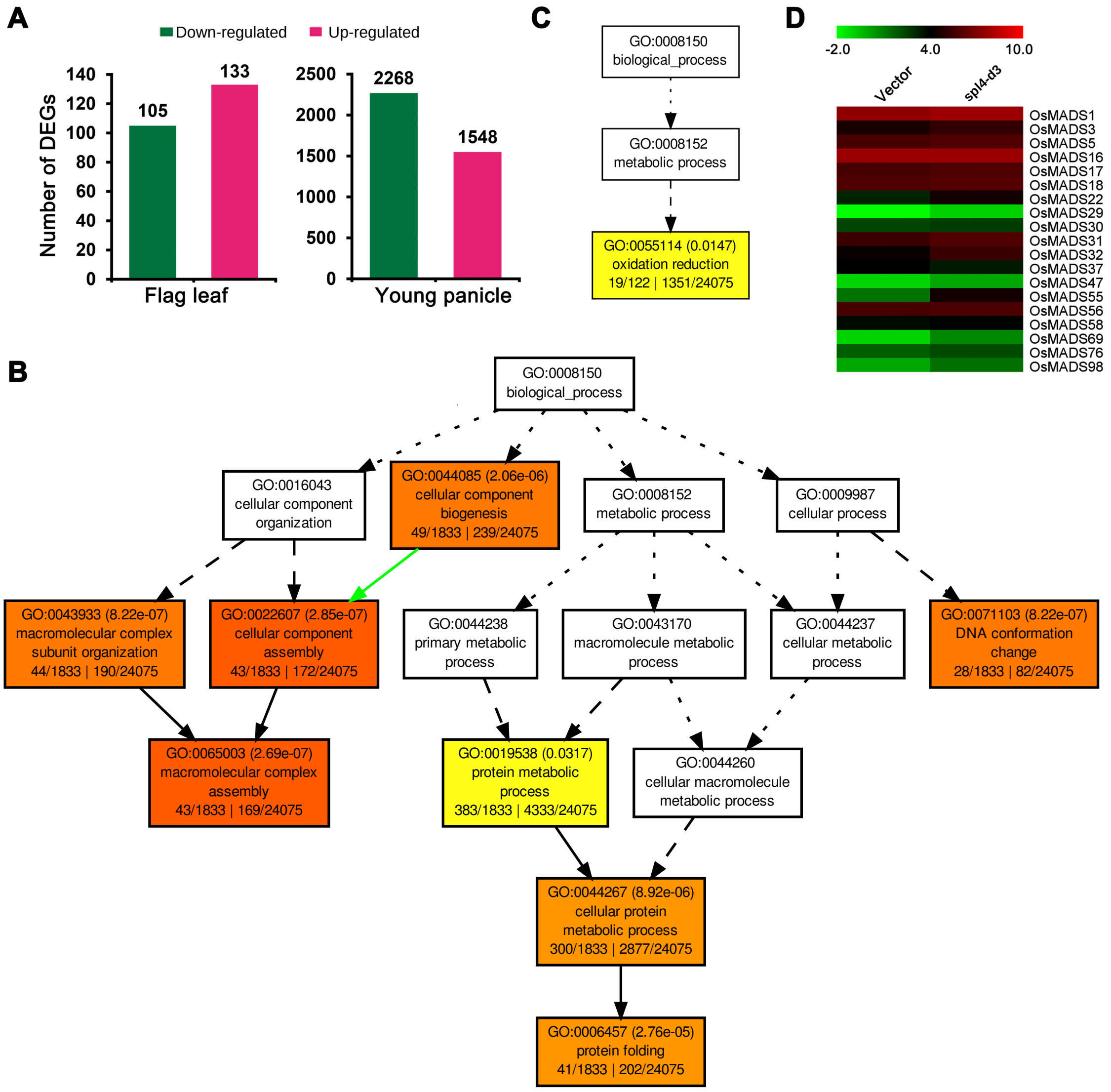

Figure S2. Differentially expressed genes (DEGs) and GO enrichment in control and *spl4-d3* using RNA-seq analysis. **(A)** Distribution of DEGs. **(B)** GO enrichment of DEGs in young panicles. **(C)** GO enrichment of DEGs in flag leaves. **(D)** Heatmaps of the expression patterns of some MADS-box genes.

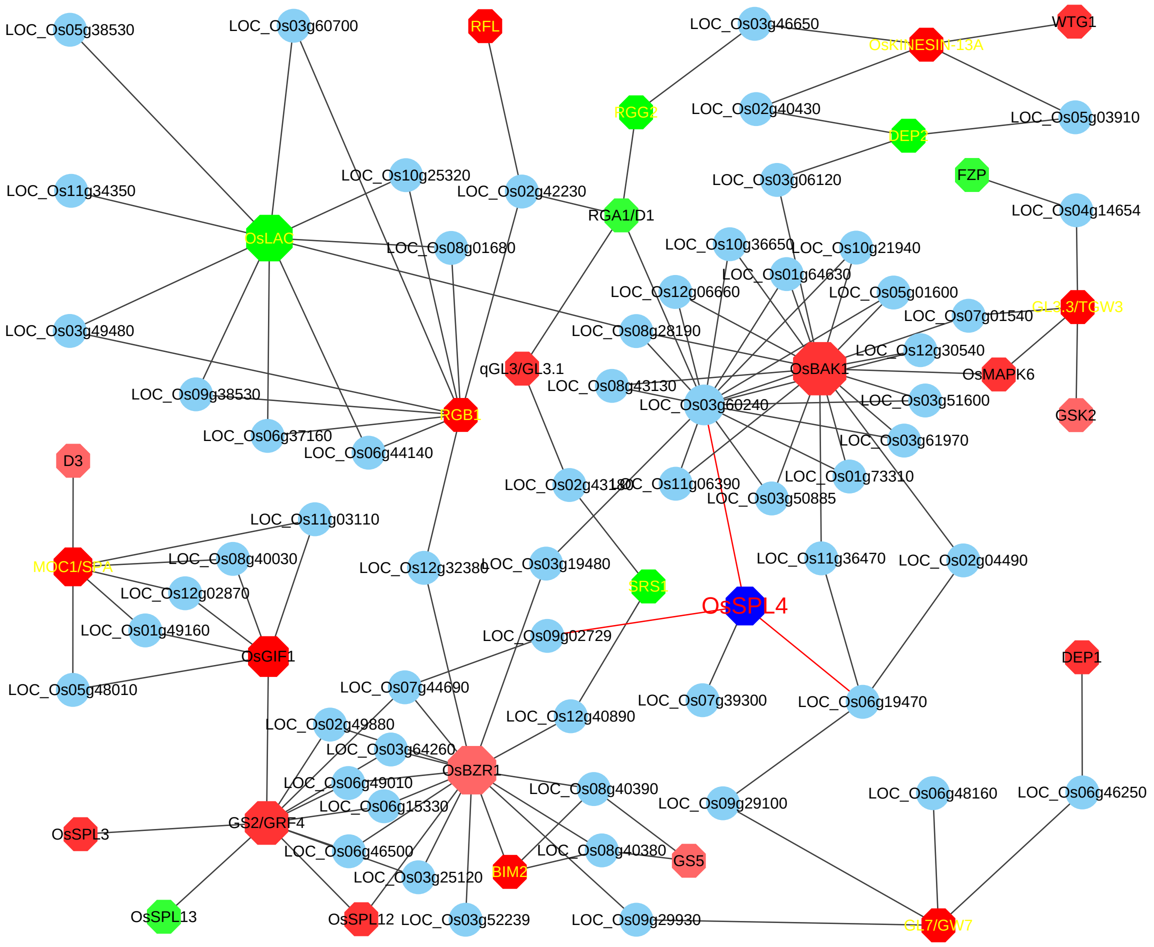

Figure S3. Co-expression network of yield related genes and *OsSPL4* in rice. The octagon represents the reported yield related genes. More number of regulated genes, larger of the octagon size. Different red color and green color indicated up-regulated and down-regulated, respectively. The octagon with yellow font were shown the significant differentially expressed yield genes. Light blue circle represent the interaction genes. *OsSPL4* gene was highlighted.

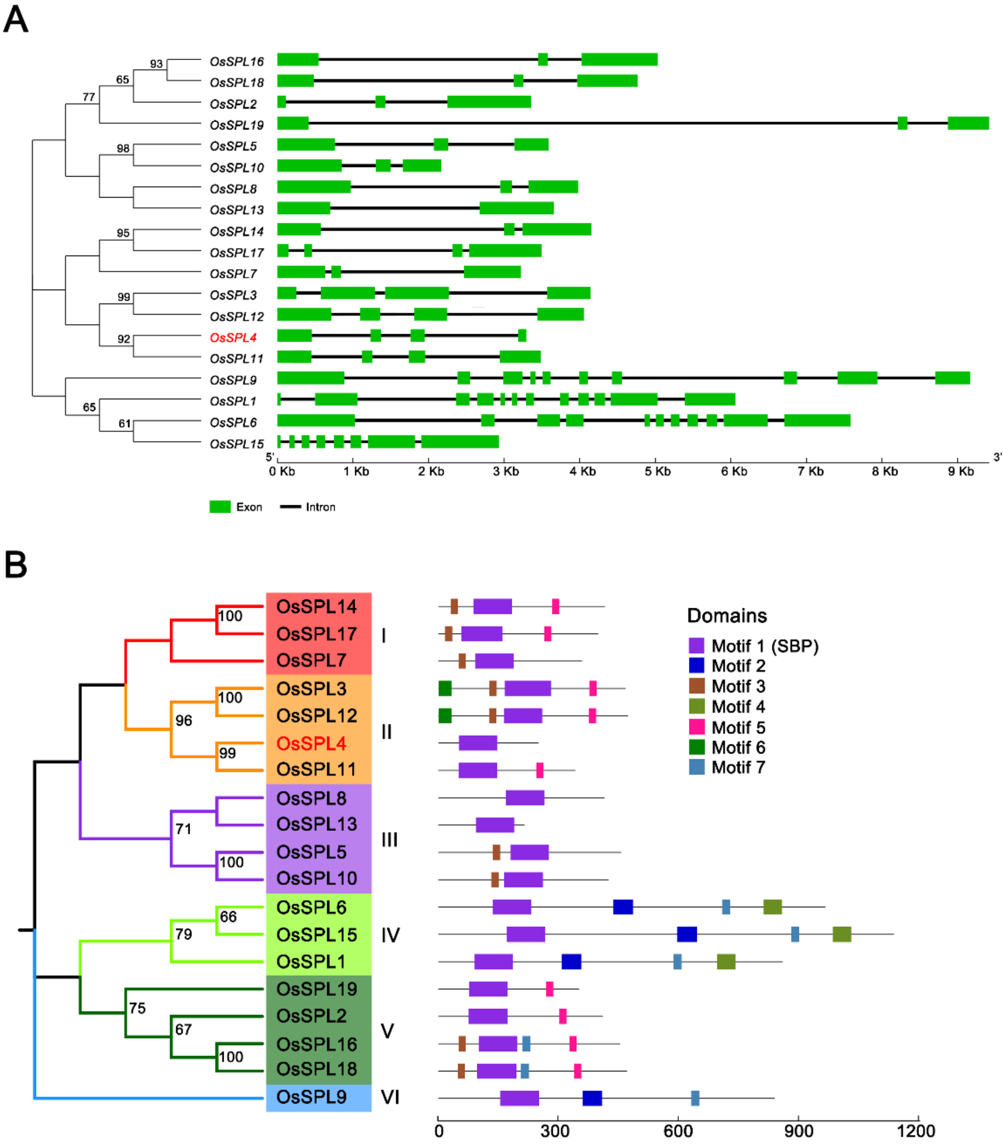

Figure S4. Phylogenetic tree of the 19 SPL family members in rice. Phylogenetic trees were constructed using MEGA 7 based on neighbor-joining method with bootstrap analysis of 1000 replicates. **(A)** Exon-intron structures of the gene sequences in the *SPL4* homologs of rice. **(B)** Protein structures and motifs of the SPL4 homologs of rice.

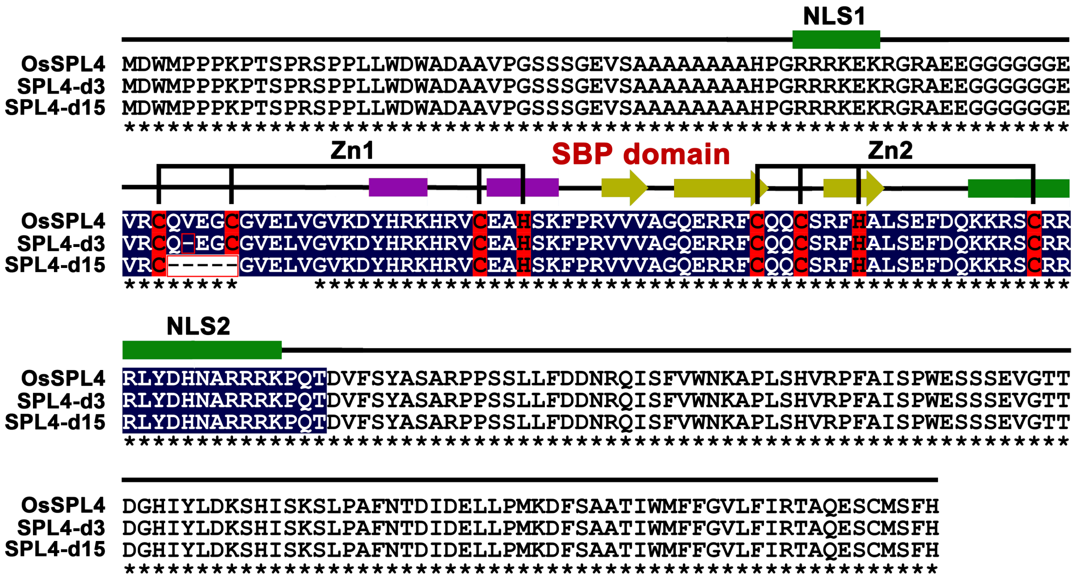

Figure S5. Protein sequences and the structures in control and the mutation lines. The SBP domain sequences were highlight in white with blue background. Purple rectangles and yellow arrows indicate the α-helices and β-sheet, respectively. The amino acids of the two zinc fingers (C3H and C2HC) were in red background. Red boxes showed the amino acid mutations of the OsSPL4 protein in the two mutants *spl4-d3* and *spl4-d15*).

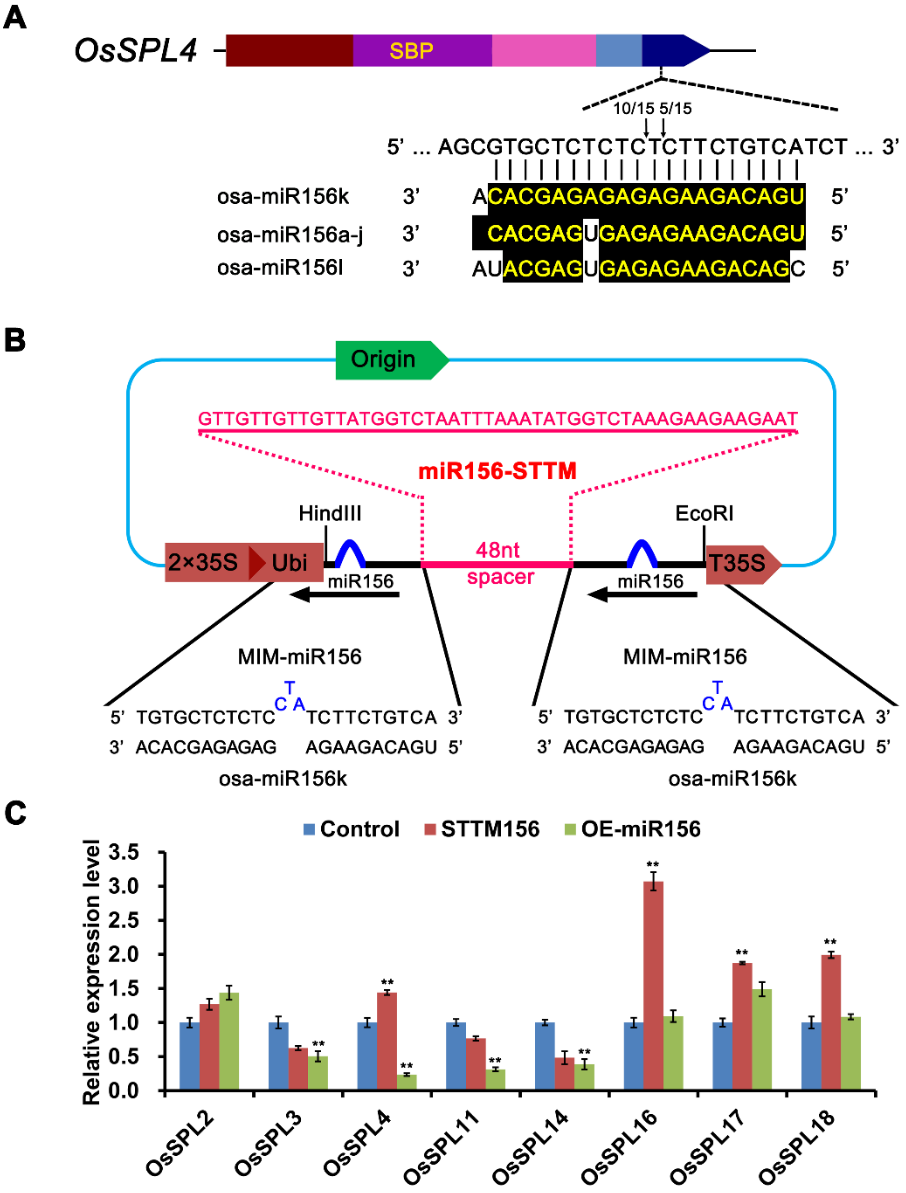

Figure S6. Schematic diagram of *OsSPL4* as the target of OsmiR156 and the vector construction of STTM156. (A) OsmiR156 targeting sequences in *OsSPL4*-miR156 and 5' RLM-RACE validation of the target site of osa-miR156. The black arrow indicates the cleavage site for osa-miR156. The rectangles indicate the exons and the blue arrow represents 3'UTR. **(B)** Schematic diagram of STTM156 vector construction. **(C)** Expression levels of the predicted miR156 targets (*OsSPL* genes) in WT, STTM156 and OE-miR156 plants performed by qRT-PCR. Significant difference between WT and STTM156 or OE-miR156 plants were determined by Student's *t*-test. Values are shown as the mean ± SD. ** $P < 0.01$, * $P < 0.05$.

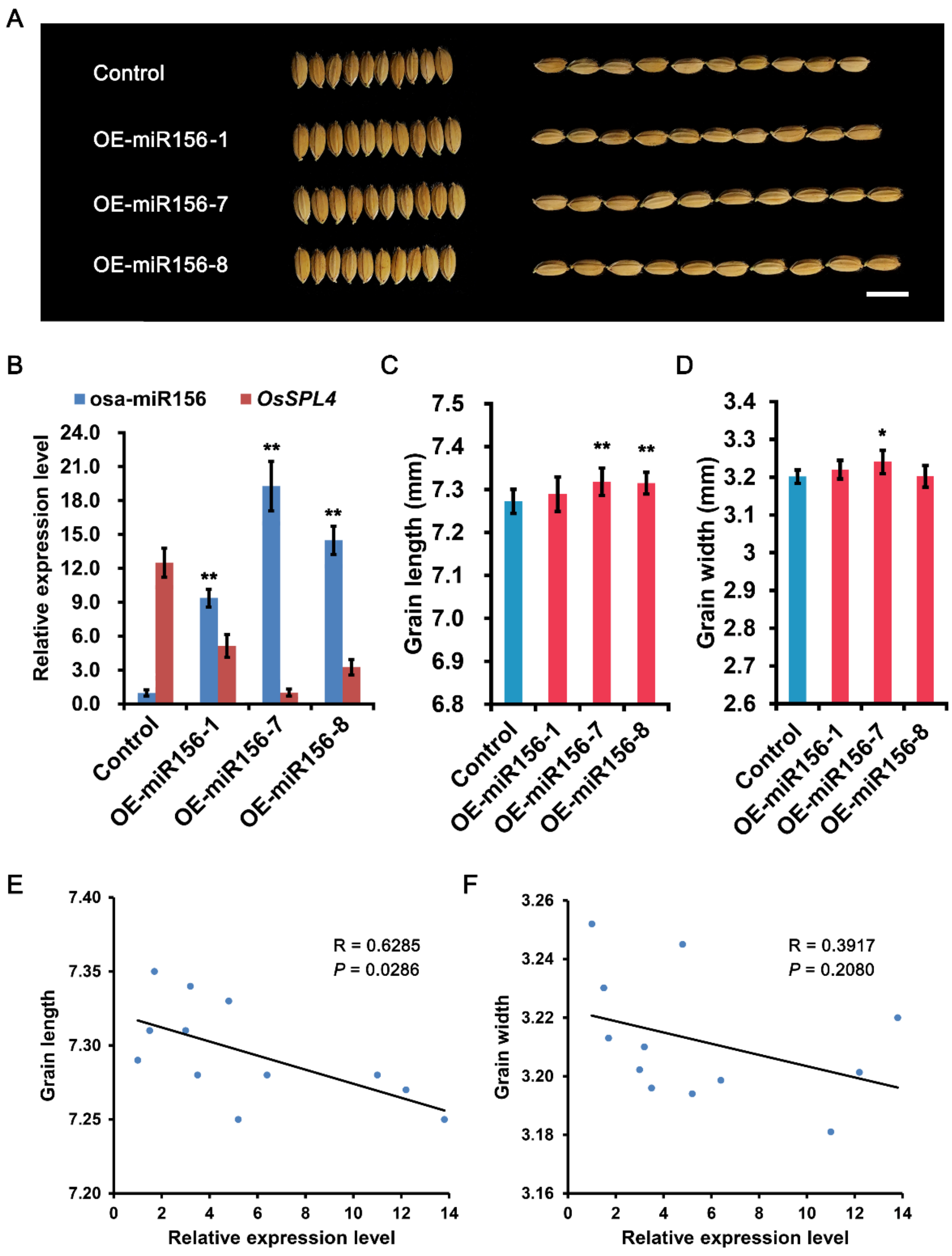

Figure S7. Overexpresssion (OE) of osa-miR156 increases rice grain length and width. (A) Grain shapes of the control and osa-miR156 OE transgenic plants, scale bar = 10 mm. **(B)** The expression level of osa-miR156 and *OsSPL4* in the osa-miR156 OE transgenic plants. **(C)** Grain length of the rice plants. **(D)** Grain width of the rice plants. Significant difference between WT and OE-miR156 plants were determined by Student's *t*-test. Values are shown as the mean ± SD. ** $P < 0.01$, * $P < 0.05$. **(E, F)** The expression level of *OsSPL4* is negatively correlated with grain length (E) and grain width (F) in OE-miR156 plants.

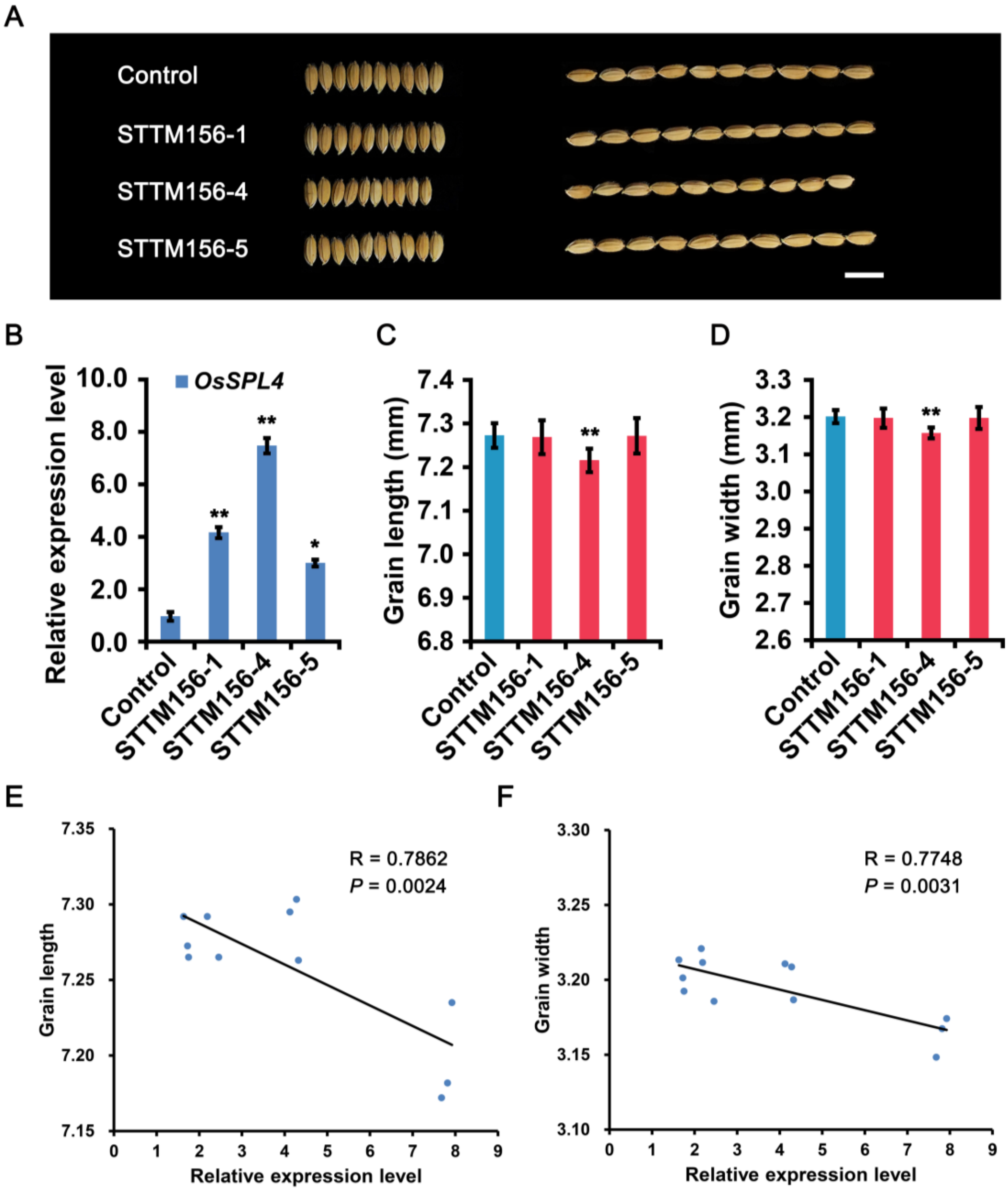

Figure S8. Osa-miR156 mimicry (STTM156) transgenic rice decreases grain length and width. (A) Grain shapes of the control and STTM156 transgenic plants, scale bar = 10 mm. **(B)** The expression level of *OsSPL4* in the control and STTM156 transgenic plants. **(C)** Grain length of the rice plants. **(D)** Grain width of the rice plants. Significant difference between WT and STTM156 plants were determined by Student's *t*-test. Values are shown as the mean ± SD. ** $P < 0.01$. **(E, F)** The expression level of *OsSPL4* is negatively correlated with grain length (E) and grain width (F) in STTM156 plants.

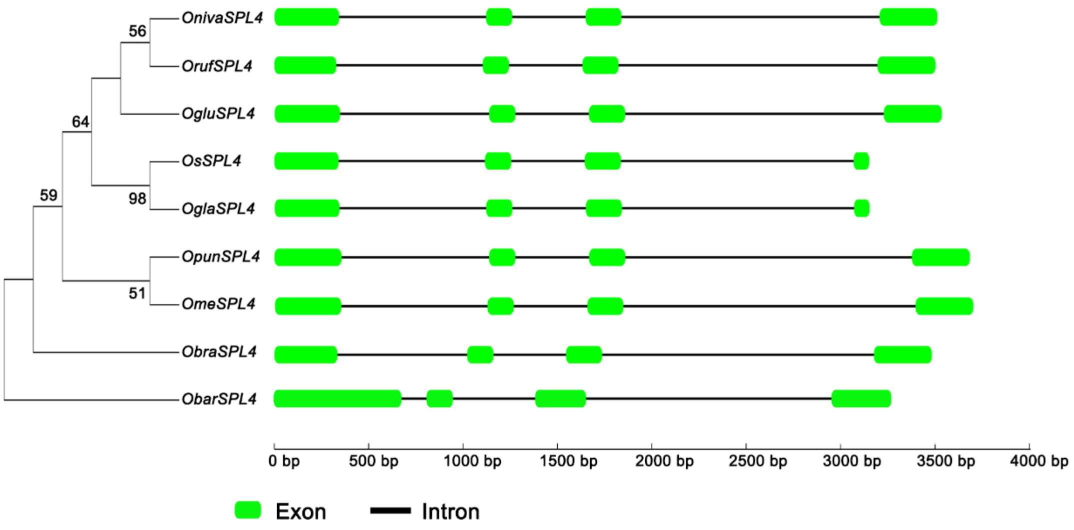

Figure S9. Phylogenetic analysis and exon-intron structures of *SPL4* orthologs in different *Oryza* genus. Green boxes indicate exons; black lines indicate introns. The length of *SPL4* genes can be estimated by the scale at the bottom. *Obar*, *Oryza barthii*; *Obra*, *Oryza brachyantha*; *Oniva*, *Oryza nivara*; *Oruf*, *Oryza rufipogon*; *Opun*, *Oryza punctata*; *Ome*, *Oryza meridionalis*; *Oglu*, *Oryza glumipatula*; *Osa*, *Oryza sativa*; *Ogla*, *Oryza glaberrima*.

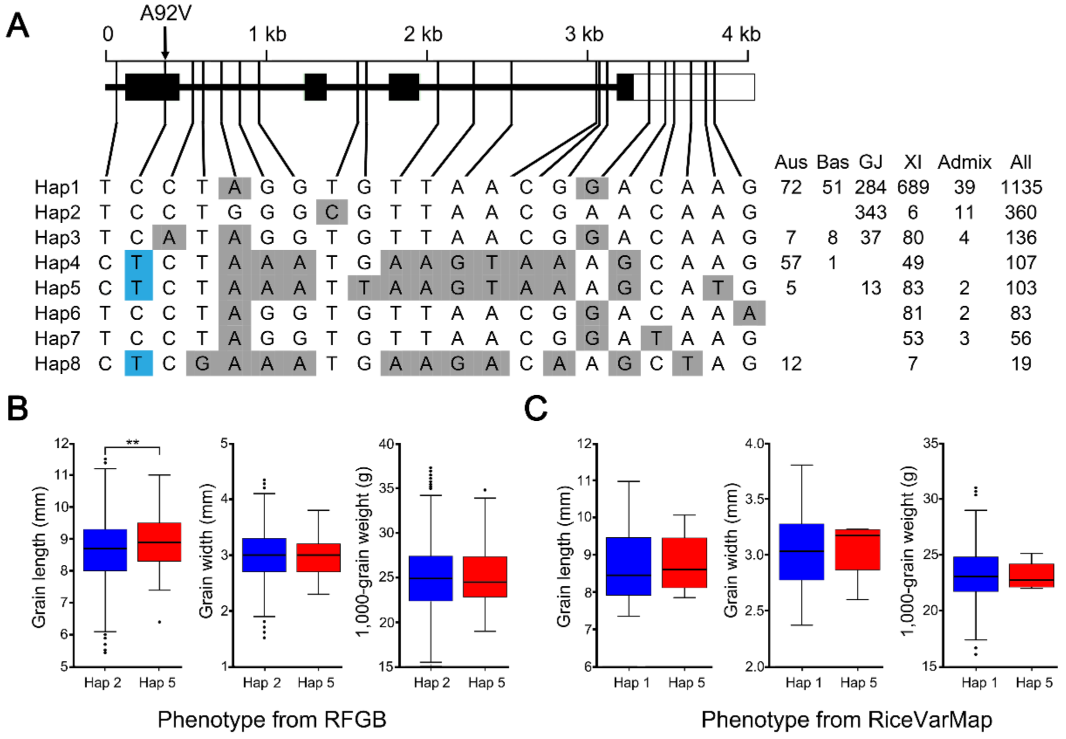

Figure S10. Different haplotypes of *OsSPL4*. (A) Eight haplotypes distributed in the 4.1 kb genomic DNA of *OsSPL4* in 2419 rice accessions. Black box represents the exon of *OsSPL4*. The arrow indicate the SNP of vg0204074278 (C-T) which lead to Ala to Val (A92V). **(B)** Grain length, width and weight difference among the 2000 rice accessions from RFGB with SNP allele (A92V). Significant difference was determined by Student's *t* test, ** $P < 0.01$. **(C)** Grain length, width and weight difference among the 529 rice accessions from RiceVarMap with SNP allele (A92V).

Table S1.   Primers used in this study.

Name	Forward primer（5' to 3'）	Reverse primer（5' to 3'）	Purpose
OsSPL4	GGCAGGTGAGGTGCCAGGTGGAA	AAACTTCCACCTGGCACCTCACC	Vector
OsSPL4-mutation	TCCCAAAATTGACCACCCAAATC	AGATAGCATCTCTGCCTCCCA	Validation
OsSPL4-OE	GCGGTACCATGGATTGGATGCCTCCTCC	GCGAGCTCTTAATGAAATGACATGCAGC	Vector
OsmiR156-OE	GCGGTACCTTGAGAGTGATGACAGAAGA	GCGAGCTCTGGACGGCAATGACAGATAG	Vector
STTM spacer	GTTGTTGTTGTTATGGTCTAATTTAAATATGGTCTAAAGAAGAAGAAT		Vector
STTM156	TGTGCTCTCTCCTATCTTCTGTCA	TGTGCTCTCTCCTATCTTCTGTCA	Vector
OsSPL4-HBT	CGGGATCCATGGATTGGATGCCTCCTCCG	GGGGTACCATGAAATGACATGCAGCTCTCC	Vector
OsmiR156	GCCGCTGACAGAAGAGAG		qRT-PCR
OsmiR156-RT	GTCGTATCCAGTGCAGGGTCCGAGGTATTCGCACTGGATACGACGTGCTC		qRT-PCR
U6	CAACGGATATCTCGGCTCT	CAACTTGCGTTCAAAGACTC	qRT-PCR
CycA3-1	AGTTTCTTCTTCCACCACTAGC	GTAGGGCTCCATGATCGAATC	qRT-PCR
CycD2-1	CCTCCTTTTTCCACTTTCCAAG	CAAGAACTCAAGAACGCATTGA	qRT-PCR
CycB1-1	GATATCGGCAACCAAGATCCAG	ATTCTGCACGACAATCTTGTTC	qRT-PCR
CycD3-1	AGGGTTCAGTCCAAGAAAAAGA	GACAAAACAGCTTCTTCCTCAC	qRT-PCR
CDKB-2	CGTCAAGATCCTGATGTACCAG	GTACTTCTTGAGAGGGACAGTG	qRT-PCR
CDKG-2	GGTTTCGGTCTGAGGATTTCTA	ACCGAAACCAAACCCTATAGAG	qRT-PCR
MCM2	ATCGGTGCTAGAAGTTATGGAG	GTAACAACACCACCAATTCGAA	qRT-PCR
MCM3	AGCATAGAAGTTCTGGAAACGA	TTGATCGAGATGATTTGCCAAC	qRT-PCR
MCM4	AGAAAGAAGGTCATAACTGCGA	TCCATATCGATTGTACCAGTGG	qRT-PCR
MCM5	GCTGCTTCATCCAAGAATACAG	GGTTTACAAGTAACACGGCAAT	qRT-PCR
OsMADS3	GAGGGGGAAGATCGAGATAAAG	GTGGATTTCACACTGTTGTTGG	qRT-PCR

OsMADS22	CCTTCGCTCGACTTGAATTTAG	GTTCATCAATGCTCAATCCCTC	qRT-PCR
OsMADS31	TGGGAGAGTAGAGCTCAAAAAG	AGAACACAATCACCCCAATTTG	qRT-PCR
OsMADS32	GAGAAACAGCTGAGGTTCATGA	AATGTTCTGGAGGTAGCAGATC	qRT-PCR
OsSPL2	AATCACCACGGAGCGGCAAGATTC	TGGTAGTACGGCTCTTGGAACACG	qRT-PCR
OsSPL3	AGCCACAACCAGAAGCAATTTCC	CTTGCCTGTTGCCTTGCATCAC	qRT-PCR
OsSPL4	TCCCAGCATTTAACACCGAC	TCCTGCGCAGTTCGGATAAA	qRT-PCR
OsSPL11	CACTACCGCGATCACTGTTTGATG	TGTCCATCACTTGGGAGAGCTG	qRT-PCR
OsSPL14	CAAGGGTTCCAAGCAGCGTAA	TGCACCTCATCAAGTGAGAC	qRT-PCR
OsSPL16	ACCGAGGAGAGCCCATACTAC	CAGAGGTGGAGCCAACGAA	qRT-PCR
OsSPL17	GCTCTGTCCACATGGTTAGGTTCC	AGCGAGAAGAAAGAGGTCCAGGTG	qRT-PCR
OsSPL18	GAAGCCACAGGCAGATAGCATGAG	ACGCGAACCTTGTCCCTTGTTG	qRT-PCR
Actin	TGCTATGTACTGCGCCATCCAG	AATGAGTAACCACGCTCCGTCA	qRT-PCR
OsSPL4-OFF1	CATGGAGGAGGACAAGAAGC	CGCAGGAGGAAGAAGTTTTG	off-target
OsSPL4-OFF2	GGTCTCAAGTTCGGCAAGAA	CAAAGCAAAAGCAGTGGTGA	off-target
OsSPL4-OFF3	GCTCAAGTTCGGCAAGAAGA	TTTGAATTTTGGCAGGAACC	off-target
OsSPL4-OFF4	TGGGGAAACGAGGATGATAG	AGTGCCCTGTGGAAGAGAAA	off-target
OsSPL4-OFF5	ACCCAAGGTTGTCATGTCCT	CATGTTTCTTGCTTGGCTGA	off-target
OsSPL4-OFF6	TGTGTCATGCAAGATCTGGTT	CACGAAAAGAAAACGCTAAAAA	off-target
GSP1	AAGGCCACCTTGGTGGATATTGGT		5'RACE
GSP2	GGGCTTTCAGTCCAGGCCTTTAGTTTATC		5'RACE

Table S2. Grain yield and associated components in the transgenic rice of *OsSPL4* in field.

**Traits**	**Control**	***spl4-d3***	***spl4-d15***	**OE1**	**OE2**
Panicle length (cm)	16.41±0.11	17.76±0.13**	17.35±0.26*	16.19±0.60	15.57±0.43
Primary panicle branches	8.63±0.45	10.27±1.08**	9.65±0.78**	8.33±1.05	7.42±0.86
Panicle number per plant	20.84±3.28	16.45±2.30*	15.32±2.15**	21.84±4.08	20.35±3.62
Grain number per panicle	58.48±4.02	68.02±5.91**	62.48±6.14*	49.64±1.80*	47.78±1.24**
Grain length (mm)	7.27±0.03	7.32±0.04**	7.36±0.06**	7.20±0.30**	7.16±0.26**
Grain width (mm)	3.20±0.02	3.30±0.04**	3.36±0.03**	3.19±0.03	3.18±0.02**
Grain thickness (mm)	2.12±0.10	2.10±0.09*	2.08±0.10**	2.13±0.08	2.14±0.09**
Grain bulk density (g/ml)	0.49±0.01	0.46±0.02**	0.47±0.01	0.50±0.02	0.51±0.01
Chalky percentage (%)	8.30±1.25	34.20±3.54**	46.80±4.32**	10.65±2.49	9.26±2.67
Chalkness degree (%)	2.70±0.95	5.54±1.34**	6.58±1.56**	3.02±1.84	3.27±1.23
1000-grain weight (g)	24.05±0.72	25.28±1.20*	24.10±1.14	23.85±0.80	23.27±0.96
Seed setting ratio (%)	72.74±2.02	75.85±2.41	73.67±2.52	70.30±2.88	61.85±2.43**
Grain yield per plant (g)	24.04±1.28	25.72±2.03**	24.36±1.82	22.91±3.25*	22.31±4.16*

Values shown are the means ± SD (n = 30 for grain yield, n = 20 for other test). Significant difference between the mutation lines or OE lines and the control plants was determined by Student's *t* test * $P < 0.05$ ** $P < 0.01$.

Table S3. Expression patterns of the floral and cell-cycle genes between control and *spl4-d3* transgenic plants.

Gene name	RGAP ID	RAP-DB ID	Control	*spl4-d3*	Fold change	log2(fold_change)	*P* value	FDR	Significant
**Floral genes**									
OsMADS1	LOC_Os03g11614	Os03g0215400	169.95	207.984	1.224	0.291363	0.13115	0.327354	
OsMADS3	LOC_Os01g10504	Os01g0201700	24.3324	36.0526	1.482	0.567225	0.0044	0.037274	**
OsMADS5	LOC_Os06g06750	Os06g0162800	51.3599	59.257	1.154	0.206344	0.32195	0.558034	
OsMADS16	LOC_Os06g49840	Os06g0712700	197.695	198.722	1.005	0.00747611	0.97015	0.98644	
OsMADS17	LOC_Os04g49150	Os04g0580700	51.8257	58.8923	1.136	0.184411	0.3378	0.573746	
OsMADS18/28	LOC_Os07g41370	Os07g0605200	57.6877	63.0197	1.092	0.127538	0.5041	0.718398	
OsMADS22	LOC_Os02g52340	Os02g0761000	9.03241	21.7021	2.403	1.26465	0.00005	0.001266	**
OsMADS29	LOC_Os02g07430	Os02g0170300	0.232451	0.514757	2.214	1.14697	1	1	
OsMADS30	LOC_Os06g45650	Os06g0667200	5.10378	6.00042	1.176	0.233498	0.5368	0.741952	
OsMADS31	LOC_Os04g52410	Os04g0614100	42.3787	61.6802	1.455	0.541466	0.00465	0.038862	**
OsMADS32	LOC_Os01g52680	Os01g0726400	18.5933	41.0254	2.206	1.14173	0.00005	0.001266	**
OsMADS37	LOC_Os08g41960	Os08g0531900	16.7872	10.425	0.621	-0.687318	0.0043	0.036611	**
OsMADS47	LOC_Os03g08754	Os03g0186600	0.489038	1.02715	2.100	1.07062	0.0723	0.228517	
OsMADS50	LOC_Os03g03100	Os03g0122600	24.1402	19.0726	0.790	-0.339939	0.1271	0.32103	
OsMADS55	LOC_Os06g11330	Os06g0217300	2.45107	21.8215	8.903	3.15427	0.00005	0.001266	**
OsMADS56	LOC_Os10g39130	Os10g0536100	50.2327	53.7242	1.070	0.0969439	0.6126	0.794002	
OsMADS58	LOC_Os05g11414	Os05g0203800	13.6097	15.2793	1.123	0.166941	0.4867	0.704597	
OsMADS69	LOC_Os08g20440	Os08g0299600	0.50253	1.72217	3.427	1.77695	0.00065	0.009877	**
OsMADS76	LOC_Os06g30830	Os06g0504450	3.46722	4.67524	1.348	0.431261	0.08715	0.256031	
OsMADS98	LOC_Os01g68560	Os01g0913900	1.03945	2.51929	2.427	1.2772	0.01025	0.065968	

Cell-cycle genes

Gene name	RGAP ID	RAP-DB ID	Control	*spl4-d3*	Fold change	Log2 (fold_change)	*P* value	FDR	Significant
CycA3-1	**LOC_Os02g39260**	**Os02g0605000**	**1.18069**	**3.79613**	**3.215**	**1.6849**	**0.00425**	**0.036293**	**
CycA3-3	LOC_Os03g11040	Os03g0208800	3.38822	3.57899	1.06	0.079025	0.7991	0.904412	
CycA3-4	LOC_Os03g11030	Os03g0208700	3.38822	3.57899	1.06	0.079025	0.7991	0.904412	
CycB1-1	LOC_Os01g59120	Os01g0805600	40.8955	43.3031	1.059	0.0825287	0.6894	0.842012	
CycB1-2	**LOC_Os05g41390**	**Os05g0493500**	**19.6755**	**32.4398**	**1.649**	**0.721363**	**0.00125**	**0.015837**	**
CycB1-3	LOC_Os01g17402	Os01g0281200	1.71532	1.93837	1.130	0.176368	0.65265	0.820345	
CycB2-1	LOC_Os04g47580	Os04g0563700	18.2956	22.4719	1.228	0.296625	0.1469	0.351003	
CycB2-2	LOC_Os06g51110	Os06g0726800	13.3259	17.4419	1.309	0.388325	0.0737	0.23133	
CycD2-1	**LOC_Os07g42860**	**Os07g0620800**	**17.7039**	**33.5917**	**1.897**	**0.924036**	**0.00005**	**0.001266**	**
CycD3-1	**LOC_Os09g02360**	**Os09g0111100**	**29.9911**	**51.1639**	**1.706**	**0.770591**	**0.0001**	**0.002279**	**
CycD4-2	LOC_Os08g37390	Os08g0479300	34.4841	37.6896	1.093	0.128237	0.4987	0.714335	
CycD5-1	LOC_Os12g39830	Os12g0588800	23.5095	26.2795	1.118	0.160696	0.4392	0.667015	
CycD5-2	LOC_Os03g42070	Os03g0617500	7.25354	10.2955	1.419	0.505252	0.0316	0.137284	
CycD5-3	LOC_Os03g10650	Os03g0203800	79.1002	63.1839	0.799	-0.324126	0.08755	0.256625	
CycT1-1	LOC_Os02g24190	Os02g0438200	12.9493	13.0821	1.010	0.0147218	0.9384	0.973229	
CDKA-2	LOC_Os02g03060	Os02g0123100	23.7297	32.9485	1.388	0.473517	0.02095	0.105162	
CDKB-1	LOC_Os01g67160	Os01g0897000	30.9687	38.863	1.255	0.327586	0.10295	0.283098	
CDKB-2	**LOC_Os08g40170**	**Os08g0512600**	**58.4161**	**120.313**	**2.060**	**1.04236**	**0.00005**	**0.001266**	**
CDKC-1	LOC_Os05g32360	Os05g0389700	12.7321	15.7343	1.236	0.305437	0.13935	0.339751	
CDKE-1	LOC_Os10g42950	Os10g0580300	26.7971	35.735	1.334	0.415263	0.03005	0.133099	
CDKF-2	LOC_Os12g23700	Os12g0424700	9.72286	12.525	1.288	0.365353	0.2353	0.466067	
CDKG-2	**LOC_Os02g39010**	**Os02g0602100**	**12.1664**	**27.8659**	**2.290**	**1.1956**	**0.0002**	**0.004009**	**
CKL3	LOC_Os01g70130	Os01g0925700	4.37913	6.94803	1.587	0.665958	0.0426	0.165277	
CKL5	LOC_Os01g27020	Os01g0367700	41.8115	42.1045	1.007	0.0100746	0.9553	0.981219	

CKL8	LOC_Os12g10190	Os12g0203000	5.55854	5.75781	1.036	0.050814	0.85715	0.933226	
E2F2	LOC_Os12g06200	Os12g0158800	24.7166	26.8068	1.085	0.117118	0.54215	0.745881	
DEL1	LOC_Os02g50630	Os02g0739700	14.2514	19.9872	1.402	0.487978	0.02725	0.124598	
DEL2	LOC_Os06g13670	Os06g0245900	5.8086	7.92325	1.364	0.447902	0.09185	0.264296	
DP2	LOC_Os10g30420	Os10g0440100	10.8165	13.3196	1.231	0.300311	0.2557	0.488629	
DP3	LOC_Os03g05760	Os03g0152100	5.64353	6.84954	1.214	0.279409	0.3195	0.555404	
MCM2	LOC_Os11g29380	Os11g0484300	15.7106	32.0034	2.037	1.02648	0.00005	0.001266	**
MCM3	LOC_Os05g39850	Os05g0476200	17.3971	31.3727	1.803	0.850667	0.00005	0.001266	**
MCM4	LOC_Os01g36390	Os01g0544450	46.3712	72.5659	1.565	0.646062	0.00555	0.044003	**
MCM5	LOC_Os02g55410	Os02g0797400	16.9489	30.6628	1.809	0.855294	0.00005	0.001266	**

Significant difference between the *spl4-d3* lines and the control plants was determined by Student's *t* test * $P < 0.05$, ** $P < 0.01$.

Table S4. KEEG pathway of the differentially expressed genes in young panicle between the *spl4-d3* and control plants.

Pathway	ID	DEGs	Background	*P* value	FDR
Metabolic pathways	ko01100	145	1243	5.29E-55	4.21348E-53
Carbon metabolism	ko01200	25	113	7.9E-16	1.30111E-14
Spliceosome	ko03040	21	134	5.23E-11	5.7226E-10
Protein processing in endoplasmic reticulum	ko04141	23	166	5.91E-11	6.39209E-10
Ubiquitin mediated proteolysis	ko04120	21	137	7.56E-11	8.05153E-10
Glycolysis / Gluconeogenesis	ko00010	14	67	3.38E-09	3.00947E-08
DNA replication	ko03030	10	36	6.07E-08	4.56218E-07
Cell cycle	ko04110	16	124	1.17E-07	8.33536E-07
Ribosome	ko03010	16	138	4.36E-07	2.84171E-06
Alanine, aspartate and glutamate metabolism	ko00250	9	35	4.88E-07	3.14944E-06
Glutathione metabolism	ko00480	10	52	1.16E-06	7.01466E-06
Glycine, serine and threonine metabolism	ko00260	9	40	1.28E-06	7.67511E-06
Peroxisome	ko04146	12	83	1.51E-06	8.98833E-06
Steroid biosynthesis	ko00100	7	20	1.68E-06	9.93003E-06
MAPK signaling pathway	ko04010	19	255	1.66E-05	7.81601E-05
Circadian rhythm	ko04710	7	31	1.93E-05	8.94869E-05
Cysteine and methionine metabolism	ko00270	8	45	2.27E-05	0.000103883
Histidine metabolism	ko00340	6	24	4.71E-05	0.000201181
Pyrimidine metabolism	ko00240	11	105	6.35E-05	0.000263415
Starch and sucrose metabolism	ko00500	8	57	0.000102	0.000403878
Proteasome	ko03050	7	44	0.000138	0.000529307
ABC transporters	ko02010	7	45	0.000156	0.000593268
Amino sugar and nucleotide sugar metabolism	ko00520	7	48	0.000223	0.000821449
Steroid hormone biosynthesis	ko00140	7	58	0.000631	0.002063549
Citrate cycle (TCA cycle)	ko00020	5	30	0.001047	0.003249397
Fatty acid metabolism	ko01212	6	48	0.001299	0.003915384
RNA degradation	ko03018	7	77	0.002846	0.007831462
Fatty acid degradation	ko00071	5	44	0.004752	0.012231251
Oxidative phosphorylation	ko00190	9	133	0.004915	0.012603948
Fatty acid biosynthesis	ko00061	3	13	0.005074	0.012876729

Table S5. The expression patterns of rice yield-related genes between the *spl4-d3* lines and control plants.

RGAP ID	Gene name	RAP-DB ID	Control	*spl4-d3*	Fold_change	log2(fold_change)	*P* value	FDR	Significant
LOC_Os06g06050	*D3*	Os06g0154200	9.587	10.933	1.140	0.1896	0.4213	0.6521	
**LOC_Os06g40780**	***MOC1/SPA***	**Os06g0610350**	**1.505**	**3.032**	**2.014**	**1.0104**	**0.0018**	**0.0203**	**
LOC_Os08g41940	*OsSPL16*	Os08g0531600	61.566	63.247	1.027	0.0389	0.8289	0.9196	
LOC_Os02g14720	*GW2*	Os02g0244100	32.054	22.779	0.711	-0.4928	0.0106	0.0676	
LOC_Os03g49880	*OsTB1/FC1*	Os03g0706500	1.285	0.746	0.580	-0.7849	0.1866	0.4058	
LOC_Os11g05470	*RCN1*	Os11g0152500	1.505	3.200	2.126	1.0883	0.1386	0.3386	
LOC_Os02g32950	*RCN2*	Os02g0531600	0.189	0.806	4.258	2.0903	0.0191	0.0988	
LOC_Os01g61480	*LAX1*	Os01g0831000	0.505	0.980	1.941	0.9566	0.1758	0.3916	
LOC_Os01g10110	*Gn1a*	Os01g0197700	0.612	1.170	1.913	0.9358	0.0827	0.2477	
**LOC_Os04g51000**	***RFL***	**Os04g0598300**	**0.853**	**10.233**	**11.994**	**3.5842**	**0.0001**	**0.0013**	
LOC_Os11g12740	*SP1*	Os11g0235200	20.401	14.312	0.702	-0.5114	0.0187	0.0974	
**LOC_Os09g26999**	***DEP1***	**Os09g0441900**	**24.463**	**35.189**	**1.438**	**0.5245**	**0.0171**	**0.0918**	
LOC_Os02g15950	*EP3*	Os02g0260200	7.445	8.462	1.137	0.1847	0.3961	0.6303	
LOC_Os02g05880	*LRK1*	Os02g0152700	27.507	21.357	0.776	-0.3651	0.0962	0.2715	
LOC_Os02g15350	*RPBF*	Os02g0252400	0.234	0.768	3.284	1.7156	0.0367	0.1508	
**LOC_Os07g47340**	***FZP***	**Os07g0669600**	**35.053**	**24.336**	**0.694**	**-0.5264**	**0.0117**	**0.0724**	
**LOC_Os03g62500**	***GL3.3/TGW3/OsSK41***	**Os03g0841800**	**116.113**	**54.467**	**0.469**	**-1.0921**	**0.0001**	**0.0013**	**
LOC_Os02g47280	*GS2/GRF4*	Os02g0701300	13.812	15.959	1.155	0.2085	0.3413	0.5778	
LOC_Os01g69830	*OsSPL2*	Os01g0922600	35.820	38.384	1.072	0.0997	0.6073	0.7903	
LOC_Os02g04680	*OsSPL3*	Os02g0139400	46.111	60.011	1.301	0.3801	0.0469	0.1748	
LOC_Os04g56170	*OsSPL8*	Os04g0656500	7.351	8.105	1.103	0.1410	0.5712	0.7660	
LOC_Os06g45310	*OsSPL11*	Os06g0663500	13.994	17.595	1.257	0.3304	0.1320	0.3285	

LOC_Os06g49010	*OsSPL12*	Os06g0703500	35.802	44.902	1.254	0.3267	0.1132	0.3002	
LOC_Os08g39890	*OsSPL14*	Os08g0509600	18.722	15.315	0.818	-0.2898	0.2570	0.4899	
LOC_Os08g41940	*OsSPL16*	Os08g0531600	61.566	63.247	1.027	0.0389	0.8289	0.9196	
LOC_Os09g32944	*OsSPL18*	Os09g0507100	55.364	48.958	0.884	-0.1774	0.3699	0.6050	
**OC_Os02g04520**	***RGG2***	**Os02g0137800**	**50.897**	**32.803**	**0.645**	**-0.6337**	**0.0023**	**0.0240**	**
LOC_Os02g45160	*OsALMT7*	Os02g0673100	1.597	2.711	1.698	0.7637	0.0626	0.2082	
LOC_Os05g09520	*GW5*	Os05g0187500	5.834	6.126	1.050	0.0706	0.8205	0.9167	
LOC_Os07g32170	*OsSPL13/GLW7*	Os07g0505200	228.679	206.329	0.902	-0.1484	0.4741	0.6938	
**LOC_Os08g38210**	***BIM2***	**Os08g0490000**	**26.348**	**41.802**	**1.587**	**0.6659**	**0.0010**	**0.0129**	**
**LOC_Os05g38420**	***OsLAC***	**Os05g0458600**	**7.027**	**3.112**	**0.443**	**-1.1751**	**0.0012**	**0.0149**	**
LOC_Os08g42540	*WTG1*	Os08g0537800	78.853	84.131	1.067	0.0935	0.6149	0.7957	
LOC_Os05g26890	*(RGA1)/DWARF1 (D1)*	Os05g0333200	52.373	41.745	0.797	-0.3272	0.1019	0.2813	
**LOC_Os03g46640**	***RGB1***	**Os03g0669100**	**45.864**	**114.484**	**2.496**	**1.3197**	**0.0001**	**0.0013**	**
LOC_Os02g54600	*SMG1*	Os02g0787300	38.535	50.810	1.319	0.3989	0.0334	0.1422	
LOC_Os06g06090	*OsMAPK6*	Os06g0154500	36.950	48.693	1.318	0.3981	0.0321	0.1385	
LOC_Os01g52050	*D61*	Os01g0718300	133.286	88.420	0.663	-0.5921	0.0152	0.0856	
LOC_Os08g07760	*OsBAK1*	Os08g0174700	44.568	48.165	1.081	0.1120	0.5498	0.7505	
LOC_Os04g39430	*D11*	Os04g0469800	7.718	12.352	1.600	0.6783	0.0114	0.0709	
LOC_Os07g39220	*OsBZR1*	Os07g0580500	225.579	227.322	1.008	0.0111	0.9550	0.9811	
LOC_Os05g06660	*GS5*	Os05g0158500	8.488	9.384	1.106	0.1447	0.5303	0.7379	
LOC_Os05g11730	*GSK2*	Os05g0207500	15.509	15.604	1.006	0.0087	0.9684	0.9857	
LOC_Os06g03710	*DLT*	Os06g0127800	17.755	20.684	1.165	0.2203	0.2630	0.4965	
LOC_Os03g44500	*qGL3/GL3.1*	Os03g0646900	22.644	27.308	1.206	0.2702	0.1481	0.3528	
LOC_Os02g51320	*OsBUL1*	Os02g0747900	67.170	53.256	0.793	-0.3349	0.0821	0.2465	
LOC_Os06g41850	*TGW6*	Os06g0623700	0.815	2.606	3.196	1.6764	0.0126	0.0757	
LOC_Os05g32270	*SMOS1*	Os05g0389000	30.225	28.140	0.931	-0.1031	0.6092	0.7917	

LOC_Os02g04130	*OsSGL*	Os02g0134200	55.312	60.519	1.094	0.1298	0.5601	0.7579	
**LOC_Os07g41200**	***GL7/GW7/SLG7***	**Os07g0603300**	**50.064**	**85.027**	**1.698**	**0.7642**	**0.0001**	**0.0023**	**
LOC_Os02g47280	*GS2/OsGRF4*	Os02g0701300	13.812	15.959	1.155	0.2085	0.3413	0.5778	
**LOC_Os03g52320**	***OsGIF1***	**Os03g0733600**	**19.374**	**67.054**	**3.461**	**1.7912**	**0.0001**	**0.0013**	**
LOC_Os06g44100	*GW6a*	Os06g0650300	0.856	2.036	2.378	1.2498	0.0367	0.1509	
LOC_Os02g13950	*FUWA*	Os02g0234200	15.281	20.391	1.334	0.4162	0.0337	0.1430	
LOC_Os05g06280	*(SAR1)/OsKINESIN-13A*	Os05g0154700	36.505	57.468	1.574	0.6547	0.0006	0.0093	**

Significant difference between the *spl4-d3* lines and the control plants was determined by Student's *t* test * $P < 0.05$, ** $P < 0.01$.

Table S6. Variations in *OsSPL4* coding region identified from RFGB Database.

Chr.	Position	Reference	Alt	Major Allele	MAF
	**SNP**				
Chr2	4073894	A	T	A	0.002
Chr2	4073898	C	A,G	C	0.022
Chr2	4073902	T	C	T	0.004
Chr2	4073905	C	T	C	0.015
Chr2	4073908	C	T	C	0.003
Chr2	4073938	C	A	C	0.003
Chr2	4073940	C	T	C	0.065
Chr2	4073963	T	C	T	0.298
Chr2	4074079	G	A	G	0.001
Chr2	4074129	G	C	G	0.006
**Chr2**	**4074278**	**C**	**T**	**C**	**0.216**
Chr2	4074397	C	A	C	0.04
Chr2	4074410	A	T	A	0.002
Chr2	4074427	G	T	G	0.001
Chr2	4074469	T	G	T	0.011
Chr2	4074517	A	G	A	0.003
Chr2	4074634	G	A	A	0.201
Chr2	4074675	T	C	T	0.018
Chr2	4074704	A	T	A	0.01
Chr2	4074710	T	A	T	0.002
Chr2	4074758	G	A	G	0.213
Chr2	4074777	G	A	G	0.021
Chr2	4074779	C	G	C	0.003
Chr2	4074808	G	A	G	0.002
Chr2	4074838	G	A	G	0.002
Chr2	4074843	G	A	G	0.002
Chr2	4074845	A	T	A	0.013
Chr2	4074863	G	A	G	0.214
Chr2	4074882	G	A	G	0.038
Chr2	4074905	G	A	G	0.022
Chr2	4074949	G	A	G	0.016
Chr2	4075017	T	C	T	0.016
Chr2	4075064	T	C	T	0.038
Chr2	4075194	C	T	C	0.006
Chr2	4075232	G	A	G	0.013
Chr2	4075475	C	T	C	0.008
Chr2	4075498	G	T	G	0.024
Chr2	4075528	T	C	T	0.014
Chr2	4075568	C	A	C	0.002

Chr2	4075593	C	T	T	0.136
Chr2	4075635	G	T	G	0.09
Chr2	4075896	C	A	C	0.038
Chr2	4075919	T	A	T	0.227
Chr2	4076100	T	C	T	0.003
Chr2	4076160	A	C	A	0.002
Chr2	4076184	T	A	T	0.226
Chr2	4076187	C	T	C	0.038
Chr2	4076201	T	C	C	0.135
Chr2	4076224	C	T	C	0.065
Chr2	4076429	A	G	A	0.214
Chr2	4076481	C	T	C	0.014
Chr2	4076499	G	A	G	0.065
Chr2	4076595	A	G	A	0.001
Chr2	4076703	G	A,T	G	0.011
Chr2	4076779	G	A	G	0.002
Chr2	4076853	A	G	A	0.002
Chr2	4076872	G	A	G	0.021
Chr2	4076901	A	T	A	0.107
Chr2	4076902	C	A	C	0.105
Chr2	4076913	C	A	C	0.004
Chr2	4076978	G	A	G	0.296
Chr2	4077123	C	T	C	0.002
Chr2	4077124	G	C	G	0.038
Chr2	4077195	C	T	C	0.001
Chr2	4077220	A	G	G	0.431
Chr2	4077306	C	T	C	0.003
Chr2	4077312	C	A	C	0.001
Chr2	4077397	C	T	C	0.016
Chr2	4077423	C	G	C	0.033
Chr2	4077465	A	G	A	0.309
Chr2	4077563	C	T	C	0.027
Chr2	4077572	G	A	G	0.065
Chr2	4077605	A	T	A	0.05
Chr2	4077654	C	T	C	0.001
Chr2	4077684	A	T	A	0.102
Chr2	4077726	G	A	G	0.033
Chr2	4077730	T	C	T	0.001
Chr2	4077777	T	C	T	0.082
Chr2	4077909	T	C	T	0.04
Chr2	4077917	T	C	T	0.032

Indel

**Chr.**	**Position**	**Reference**	**Alt**	**MAF**

Chr2	4073894	A	ACTT	0.296
Chr2	4073895	C	CTTG	0.227
Chr2	4073902	T	TTCTTCCTCC,TTCTTCC,TTCTTCTTCC	0.191
Chr2	4074106	TCCGCCGCCGCCG	TCCGCCG,T,TCCGCCGCCGCCGCCGCCG	0.228
Chr2	4074109	G	GCCGCCT,GCCA	0.101
Chr2	4074118	G	GAGGTAT	0.244
Chr2	4074119	C	CT	0.085
Chr2	4074128	C	CCGCCGT,CCGCCGC,CCGT	0.101
Chr2	4074380	C	CCT	0.012
Chr2	4074396	G	GAAA,GCAAAAAAAAAA	0.116
Chr2	4074397	C	CA,CAA,CAAA	0.271
Chr2	4074410	A	AT	0.024
Chr2	4074511	A	AT	0.002
Chr2	4074526	T	TTCC	0.001
Chr2	4074720	GTT	GTTT,G,GT	0.133
Chr2	4075029	C	CT,CTTT,CTT	0.116
Chr2	4076175	G	GA	0.038
Chr2	4076787	GAA	G,GA	0.135
Chr2	4076854	A	AATTT	0.029
Chr2	4077731	A	AACCTGTGATTGTCCC	0.001
Chr2	4077732	AT	ATT,A	0.053

Table S7. Variations in *OsSPL4* coding region identified from RiceVarMap Database.

Variation ID	Chr.	Position	Primary Allele	Secondary Allele	Primary Allele Frequency	Variation	Impact of variations
vg0204074106	Chr2	4074106	T	TCCGCCG CCGCCG	53%	TCCGCCGCCG CCG → TCCGCCGCCG CCGCCGCCGC CGCCG,T,TC CGCCG,TCCG CCGCCGCCGC CGCCG,TCCG CCGCCGCCGC CG	inframe_deletion
vg0204074109	Chr2	4074109	G	GCCA	99%	G → GCCGCCT,GC CA	inframe_insertion
vg0204074128	Chr2	4074128	C	CCGCCGT	94%	C → CCGCCGT,T	missense
vg0204074118	Chr2	4074118	G	A	98%	G→A	missense
**vg0204074278**	**Chr2**	**4074278**	**C**	**T**	**79%**	**C→T**	**missense**
vg0204075194	Chr2	4075194	C	T	99%	C→T	missense
vg0204075232	Chr2	4075232	G	A	99%	G→A	synonymous

Table S8. Phenotypes for the different haplotypes in *OsSPL4* of rice (included as a separate excel file).
